# Galactic cosmic radiation exposure causes multifaceted neurocognitive impairments

**DOI:** 10.1007/s00018-022-04666-8

**Published:** 2023-01-06

**Authors:** Yasaman Alaghband, Peter M. Klein, Eniko A. Kramár, Michael N. Cranston, Bayley C. Perry, Lukas M. Shelerud, Alice E. Kane, Ngoc-Lien Doan, Ning Ru, Munjal M. Acharya, Marcelo A. Wood, David A. Sinclair, Dara L. Dickstein, Ivan Soltesz, Charles L. Limoli, Janet E. Baulch

**Affiliations:** 1 Department of Radiation Oncology, Medical Sciences I, University of California Irvine, Room B-146D, Irvine, CA 92697-2695 USA; 2 Department of Neurosurgery, Stanford University, Palo Alto, CA 94305 USA; 3 Department of Neurobiology and Behavior, School of Biological Sciences, University of California, Irvine, 92697-2695 USA; 4 Center for the Neurobiology of Learning and Memory, University of California, Irvine, 92697-2695 USA; 5 Institute for Memory Impairments and Neurological Disorders, University of California, Irvine, 92697-2695 USA; 6 Department of Pathology, Uniformed Services University of Health Sciences, Bethesda, MD 20814 USA; 7 The Henry M. Jackson Foundation for the Advancement of Military Medicine (HJF), Bethesda, MD 20817 USA; 8 Department of Genetics, Blavatnik Institute, Paul F. Glenn Center for Biology of Aging Research, Harvard Medical School, Boston, MA 0211 USA; 9 Department of Anatomy and Neurobiology, University of California, Irvine, 92697-2695 USA; 10 Department of Neurology and Neurological Sciences, Stanford University, Palo Alto, CA 94305 USA

**Keywords:** Cognitive dysfunction, Electrophysiology, Synaptic plasticity, Space radiation

## Abstract

Technological advancements have facilitated the implementation of realistic, terrestrial-based complex 33-beam galactic cosmic radiation simulations (GCR Sim) to now probe central nervous system functionality. This work expands considerably on prior, simplified GCR simulations, yielding new insights into responses of male and female mice exposed to 40–50 cGy acute or chronic radiations relevant to deep space travel. Results of the object in updated location task suggested that exposure to acute or chronic GCR Sim induced persistent impairments in hippocampus-dependent memory formation and reconsolidation in female mice that did not manifest robustly in irradiated male mice. Interestingly, irradiated male mice, but not females, were impaired in novel object recognition and chronically irradiated males exhibited increased aggressive behavior on the tube dominance test. Electrophysiology studies used to evaluate synaptic plasticity in the hippocampal CA1 region revealed significant reductions in long-term potentiation after each irradiation paradigm in both sexes. Interestingly, network-level disruptions did not translate to altered intrinsic electrophysiological properties of CA1 pyramidal cells, whereas acute exposures caused modest drops in excitatory synaptic signaling in males. Ultrastructural analyses of CA1 synapses found smaller postsynaptic densities in larger spines of chronically exposed mice compared to controls and acutely exposed mice. Myelination was also affected by GCR Sim with acutely exposed mice exhibiting an increase in the percent of myelinated axons; however, the myelin sheathes on small calibur (< 0.3 mm) and larger (> 0.5 mm) axons were thinner when compared to controls. Present findings might have been predicted based on previous studies using single and mixed beam exposures and provide further evidence that space-relevant radiation exposures disrupt critical cognitive processes and underlying neuronal network-level plasticity, albeit not to the extent that might have been previously predicted.

**Supplementary Information:**

The online version contains supplementary material available at 10.1007/s00018-022-04666-8.

## Introduction

On Earth and in low Earth orbit astronauts are protected from radiation exposure by the Earth’s magnetosphere. However, as we now look to carry out deep space exploration to the Moon and to Mars significant concerns remain regarding the detrimental health effects of exposure to galactic cosmic radiation (GCR). The most alarming of these concerns may be the effects of space radiation exposure on the central nervous system (CNS) [1]. GCR is comprised primarily of protons and helium nuclei, with the addition of highly energetic heavy nuclei known as HZE particles [high (H) atomic number (Z) and energy (E)]. Current shielding cannot prevent these charged particles from penetrating the hulls of spacecraft and exposing the human body. Estimates suggest that astronauts will be exposed to approximately 13 cGy of GCR during each year of a mission, with the bulk of the exposure occurring en route to Mars [1, 2]. A large body of research using rodent models supports the hypothesis that exposure to these energetic charged particles elicit impairments in learning and memory and elevate anxiety and depression [3–8]. However, one caveat associated with those studies is that they have typically evaluated the effect of a single ion or up to 6 ion sequential exposures that do not represent the full complexity of the multiple ions and energies that define the actual GCR spectrum. While simplified GCR simulations (GCR Sim) using 5–6 beams including protons, ^28^Si, ^4^He, ^16^O, ^56^Fe provide more realistic scenarios of the space radiation environment, they still fall short of representing the complex mixture of particles to which astronauts will be exposed during Mars missions.

Until recently, technological limitations have prevented evaluation of more space-relevant combinations of radiation exposures on CNS function. However, previous work from our laboratory and from the laboratories of others identified risks to the CNS following exposure to simplified 5- or 6- beam GCR simulations [3, 4, 9]. To more accurately recapitulate the space radiation environment, NASA has now developed the capability to deliver a complex GCR Sim that includes 33 distinct beams, predominated by protons and ^4^He particles of various energies, interspersed with infrequent beams of different heavy ions [10]. Another shortcoming in the majority of past studies analyzing the impact of GCR exposures on the CNS was that the radiation doses were delivered at excessive total doses and dose rates. While the dose rates with which the complex GCR Sim beams are delivered in our current study are still greater than those that will be encountered during deep space travel, very low daily doses were delivered, allowing for a much more realistic overall representation of the CNS effects expected to occur due to radiation exposures during deep space travel. There has been one prior report utilizing this optimized complex GCR Sim [11] that found minimal change in exploratory and object recognition-based tasks in male mice, but did find deficits in sociability behaviors. While these studies pointed to GCR induced cognitive decrements, mechanistic studies were not conducted and the radiation countermeasures used were not impactful on outcomes.

Previous work from our laboratory and from the laboratory of others identified risks to the CNS following exposure to simplified 5- or 6-beam GCR simulations [3, 4, 9]. The goal of our current study was to use this same realistic 33-beam GCR Sim exposure model as that used by Kiffer and colleagues [11] at a more space-relevant total dose, delivered either chronically or acutely, to gain an improved understanding of the CNS impairments that astronauts may face during deep space exploration. Furthermore, we conducted an extensive longitudinal behavioral battery with both male and female mice, with follow-up electrophysiology and structural determinants of radiation injury in male mice to garner a deeper mechanistic insight of space radiation injury to the brain. Importantly, the focus of these studies was not designed to evaluate dose response or dose rate effects, nor differences between the acute and chronic GCR Sim paradigms, but rather, to elucidate whether these complex mixtures of ions and ion energies could disrupt critical cognitive processes and underlying neuronal network plasticity. However, these impairments may be less severe than those observed in previous acute space radiation studies of the CNS, suggesting that the hazards of deep space radiation exposures to astronaut CNS capabilities may not be as detrimental as might have been predicted by studies evaluating more simplified irradiation paradigms. Nonetheless, clear evidence of cognitive deficits arising in both male and female animals, regardless of the time course of GCR Sim exposure, still implicates certain CNS risks associated with the space radiation environment. The focus of these studies was to elucidate whether complex mixtures of particles and particle energies could disrupt critical cognitive processes and underlying neuronal network plasticity and whether those disruptions were more or less severe than those observed in previous space radiation studies of the CNS. Present findings provide a more realistic context for establishing whether the hazards of deep space radiation exposures represent a threat to astronaut performance during a Mars mission.

## Materials and methods

### Animals and irradiations

All animal experimentation procedures described in this study are in accordance with the guidelines provided by NIH and approved by all Institutional Animal Care and Use Committees (IACUC) and performed within institutional guidelines. Single cohorts of 178 wild-type male and 91 female mice (C57BL/6J, JAX, Bar Harbor ME) were acclimatized and aged in the NASA Space Radiation Laboratory (NSRL) at Brookhaven National Laboratory (Upton, NY) for a minimum of 2 months prior to initiation of the study. The mice were group-housed under standard conditions (20 °C ± 1 °C; 70% ± 10% humidity; 12 h:12 h light and dark cycle) and provided ad libitum access to food and water.

**Fig. 1:**
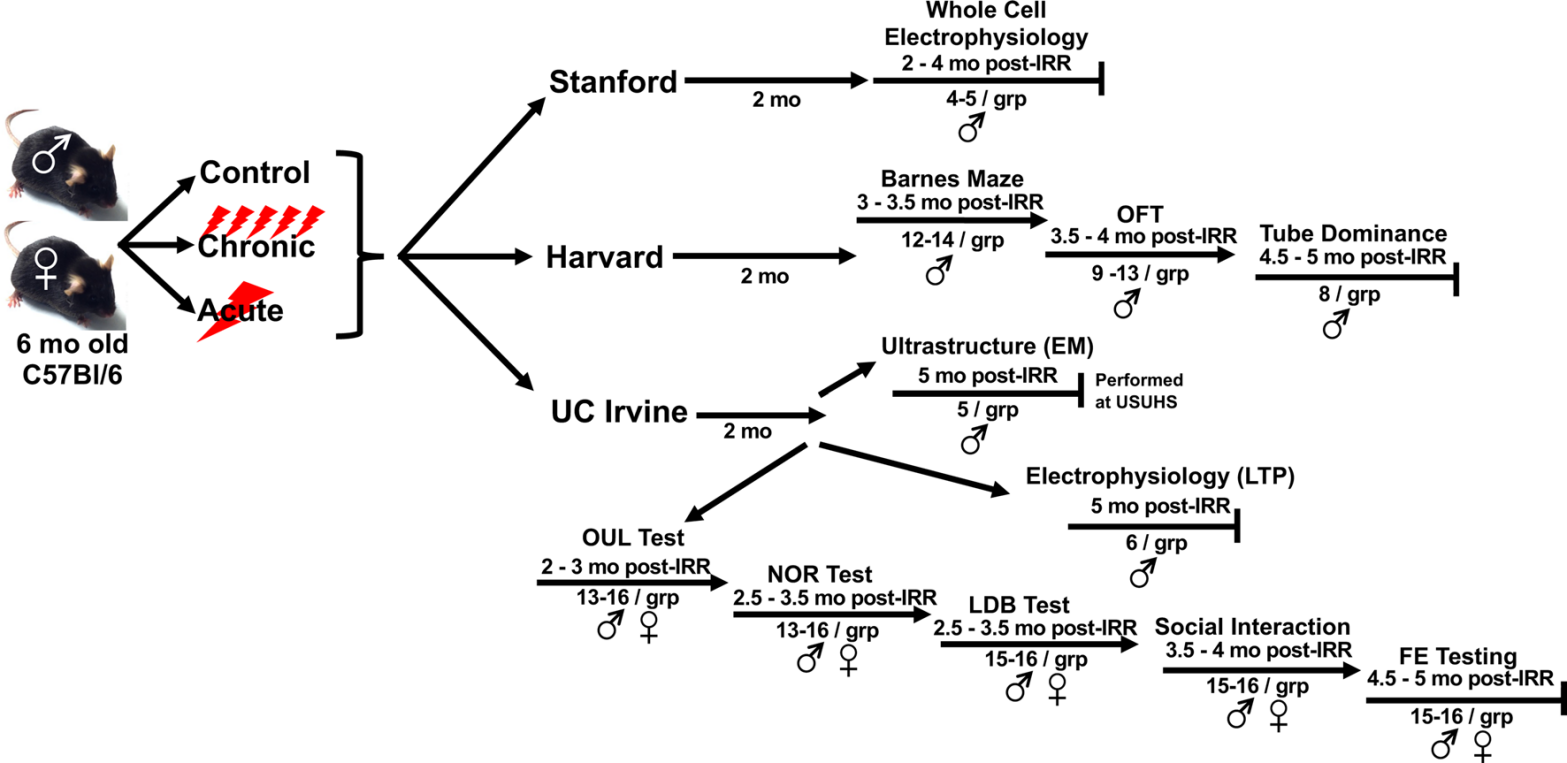
Study design. Single cohorts of 178 wild-type and 91 female C57BL/6J were randomly divided into 3 experimental groups: sham irradiated controls, and acutely or chronically irradiated using the 33-beam GCR Sim protocol [59–60 male mice and 30–31 female mice for each irradiation paradigm]. Chronically irradiated animals received a GCR Sim dose of 2.08 cGy/day, 6 days/week for 4 weeks (24 irradiation days; total accumulated dose ~ 50 cGy). Acutely irradiated animals received a single total GCR Sim dose on same day that the chronically irradiated mice received their final exposure (~ 40 cGy over ~ 2 h). Within 5 days post-irradiation mice were shipped to their respective institutions (i.e., Harvard, UC Irvine, Stanford). Animals were acclimated at least 2 months prior to behavioral, electrophysiological or structural analyses. (IRR, irradiation; OFT, open-field testing; EM, electron microscopy; USUHS, Uniformed Services University of Health Sciences; LTP, long-term potentiation; OUL, object in updated location; NOR, novel object recognition; LDB, light–dark box; FE, fear extinction)

Mice were irradiated using the NASA developed 33-beam GCR simulation (GCR Sim) protocol [10], starting at 6 months of age, during the NSRL experimental cycle 19B. Male and female mice were each randomly divided into 3 experimental groups: sham irradiated controls, and acutely or chronically irradiated using the 33-beam GCR Sim protocol (59–60 male mice and 30–31 female mice for each irradiation paradigm). The 33 charged particle species were delivered in rapid succession to simulate the spectrum of radiations experienced during a deep space mission while inside a spacecraft [2, 12, 13] and were delivered with the order, energies and doses as described by Simonsen and colleagues [10]. The NSRL physics staff performed all radiation dosimetry and confirmed spatial beam uniformity. Chronically irradiated animals received a GCR Sim dose of 2.08 cGy/day, 6 days a week for 4 weeks, for a total of 24 irradiation days and a total accumulated dose of ~ 50 cGy. Acutely irradiated animals received a single total GCR Sim dose of ~ 40 cGy over a duration of ~ 2 h on same day that the chronically irradiated mice received their final exposure. Further details of the animal irradiations, numbers of mice per treatment and sex, and experimental flow are given in Fig. 1.

### Cognitive testing

To determine the effects of chronic and acute GCR on cognitive function, mice were subjected to a range of behavioral tests. Concurrent behavioral testing of control and irradiated mice occurred across 3 months, beginning 2 months after the conclusion of GCR exposures. Data analysis was conducted independently and blindly and is presented as the average of all trials scored for each task. All behavioral testing was conducted following previously published and carefully controlled protocols (SI; 14).

### Extracellular field recordings and whole cell electrophysiology

Hippocampal slices for extracellular field recording and whole cell electrophysiology experiments were prepared as previously described and are detailed in the SI [3, 15]. Extracellular field recordings were performed for both male and female mice at 5 months after the completion of irradiations. Based on the labor-intensive nature of whole cell electrophysiology, only male mice were evaluated 2–4 months after the completion of irradiation using a separate group of mice.

### Quantitative analysis of synapses and myelin

Brains from male mice were dissected into the CA1 region of the hippocampus, the medial prefrontal cortex (mPFC) and the corpus callosum at 5 months after the completion of irradiations. Brain regions were sectioned and prepared for electron microscopy (EM) experiments as previously described and are detailed in the SI [16, 17]. As for the whole cell electrophysiology, only male mice were evaluated by EM as informed by the more robust effect of GCR Sim exposure on extracellular field recordings for male mice given the equally labor-intensive nature of structural EM analyses.

### Statistical analyses

Statistical analyses for behavioral, extracellular field recording and EM experiments were carried out using GraphPad Prism (v8) software. For the OUL, NOR, LDB and SIT assays, following confirmation of normal Gaussian distribution of behavior data one-way ANOVAs were used to assess significance between control and irradiated groups, and when overall group effects were found to be statistically significant, a Bonferroni’s post hoc test was used to compare chronic and acute GCR groups against the control group. For these behavior tests, an outlier was defined as a mouse whose behavior was outside of 2 standard deviations of the mean and was excluded from the analysis. Unless stated otherwise, behavior data were expressed as mean ± SEM and all analyses considered a value of *P* $<$ 0.05 to be statistically significant.

For tube dominance, an unpaired Student’s *t*-test was conducted. Extracellular field recording measurements were analyzed using one-way ANOVA followed by a Dunnett’s multi-comparison test or two-way ANOVA followed by a Bonferroni’s post hoc test. For synapse characterization and percent myelinated axons, a one-way ANOVA were performed followed by Bonferroni’s multiple comparison test.

To account for the nested data produced by whole cell electrophysiology experiments, differences between treatment groups were evaluated using a linear mixed-effect model (LMM) regression analysis approach [18]. LMMs were fit in R using the lme4 package [19], where outcome measures were analyzed against treatment fixed effects and a random effect combining the nested variation from multiple cell recordings per animal. A Satterthwaite-based *F*-test performed with the pbkrtest package [20] was used to evaluate the main effect of treatment against a null LMM fit lacking the treatment term, followed by Tukey’s HSD post hoc testing. The Satterthwaite method provides effective degrees of freedom accounting for variances within the LMM fit. Calculation of estimation statistics-based confidence intervals was performed with the DABEST package in Python [21]. Cumming estimation plots include a 5000 resampling, bias-corrected and accelerated bootstrap analysis to determine the nonparametric confidence interval of differences between groups. We quantified effect sizes with an unbiased Cohen’s *d* test. ANOVA measures were used for AP frequency measurements that spanned many intervals. Statistical analysis was performed in Python or R. Unless stated otherwise, results were expressed as mean ± SEM and all analyses considered a value of *P* $<$ 0.05 to be statistically significant.

To account for the nested data produced by g-ratio quantification, differences between treatment groups were evaluated using a linear mixed-effect model (LMM) regression analysis approach. LMMs were fit in R 4.1.2 [22] using the lme4 [19] and lmerTest [23] packages, where outcome measures were analyzed against treatment fixed effects and a random effect for animal ID, representing the nested variation from multiple synapse or axon measurements per animal. Significant interaction effects were decomposed by comparison of estimated marginal means with the demeans package in R [24]. Results were expressed as mean ± SEM and all analyses considered a value of *P* $<$ 0.05 to be statistically significant.

## Results

### GCR Sim exposure results in sex-specific impairments in cognitive domains and anxiety paradigms

To evaluate the effects of chronic or acute 33-beam complex GCR Sim exposures, delivered across either acute or chronic time courses, we employed our extensive behavioral testing platform beginning 2 months after the completion of irradiations with completion of testing occurring at 5 months post-irradiation. Both male and female mice were used to determine whether these distinct exposure paradigms result in sex-specific impairments in cognition and anxiety-like behavior. To this end, we utilized behavioral tasks with a specific emphasis on the hippocampus–medial prefrontal cortex (mPFC) neural circuits. Collectively, our data show that chronic and acute GCR exposures differentially affect female and male mice on particular behavioral tests including spontaneous exploration, anxiety test assays and social interactions. Further, electrophysiological recordings using male mice identify cellular-level alterations in hippocampal neuron function and associated disruptions in network-level synaptic plasticity.

**Fig. 2:**
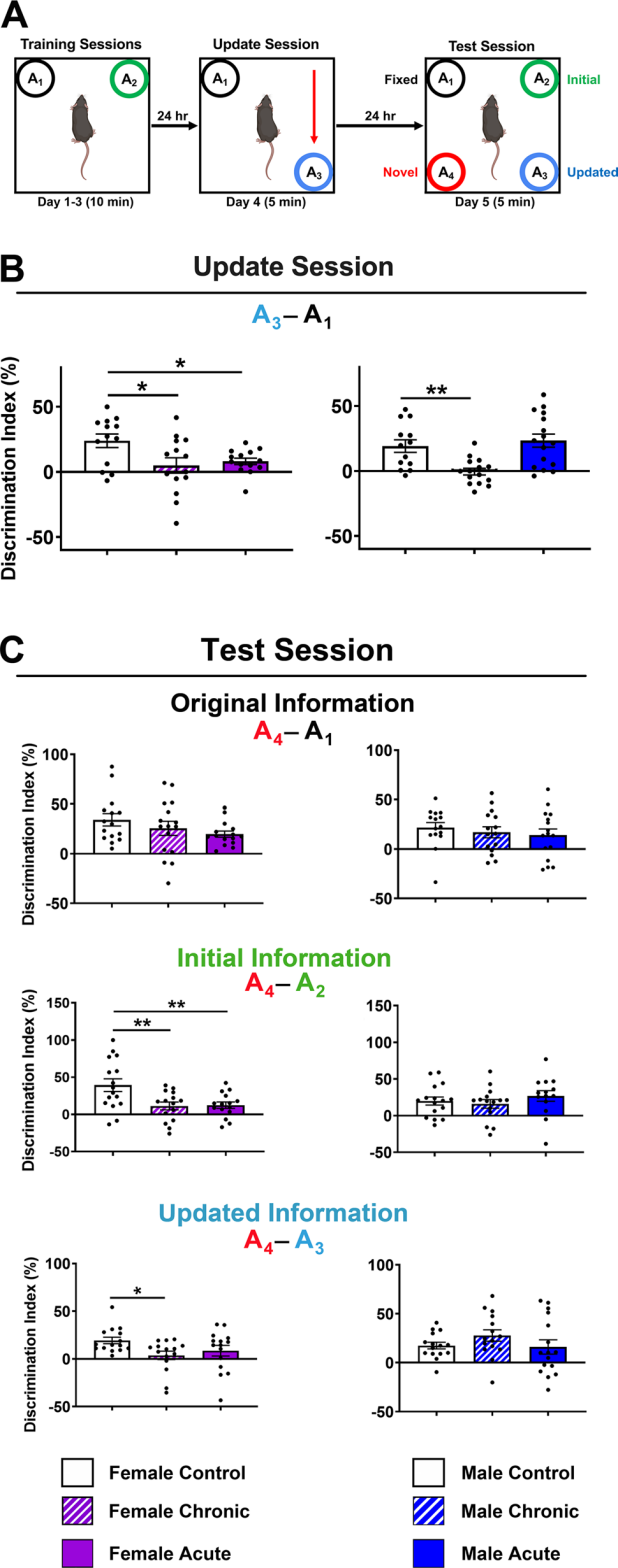
Exposure to simulated GCR elicits impairments in memory formation and updating. **A** Experimental design. All objects were identical aside from location. **B** Female mice exposed to chronic or acute GCR Sim exhibited significantly lower discrimination indices (DI) relative to controls during the update session, demonstrating no preference for the object in the updated location (A_3_) as compared to the fixed location object (A_1_). Only chronically irradiated male mice were impaired in update session performance. **C** During the test session, female and male mice exposed to either GCR Sim paradigm retained the memory of the fixed location (A_1_) relative to the novel location (A_4_), exhibiting DI scores similar to control animals (top panels). The irradiated male mice also retained the memory of the initial location (A_2_) relative to the novel location (A_4_), but both chronically and acutely irradiated females showed significantly lower DIs relative to control animals (middle panels). Similarly, irradiated male mice retained the memory of the updated location (A_3_) relative to the novel location (A_4_), but the chronically irradiated female mice showed significantly lower DIs relative to control animals. Data are mean ± SEM (*N* = 13–16 per group); *P* values derived from one-way ANOVA followed by a Bonferroni’s multiple comparison test. *P < 0.05, **P < 0.01

The object in updated location (OUL) task is a memory updating paradigm that assesses both the original memory and the updated information in a single test session [25]. Further, the OUL task uses incidental learning that takes advantage of the innate preference of rodents for novelty. Following initial habituation to the arena, mice learned the locations of 2 identical objects in a familiar context (Fig. 2A; training session, days 1–3). The following day, during the update session (day 4), all animals were exposed to one familiar fixed object location (A_1_) and one identical object moved to a new updated location (A_3_). Control animals were shown to have successfully acquired the original object location memory (OLM) during the update session, recognizing the A_3_ location as the novelty, as did the acutely irradiated male mice (Fig. 2B). However, acute and chronically irradiated females (*F*_(2,38)_ = 4.48; *P* = 0.018) and the chronically irradiated males exhibited a lack of preference for the object in the novel updated A_3_ location (*F*_(2,41)_ = 9.001; *P* = 0.0006).

The day after the update session, all groups were given a test session (day 5) (Fig. 2C). At test, memory for the updated information was examined via comparison between exploration of the object in the novel location (A_4_) to exploration of the fixed location (A_1_), the initial location (A_2_) and the updated location (A_3_). As cognitively intact mice prefer novelty, intact memory for the original training session or the updated information is demonstrated by preferential exploration of the object in the novel location (A_4_) compared to each of the other objects as indicated by a higher score on the discrimination index (DI; see methods). Female GCR Sim-exposed mice retained a similar preference as control animals for the object in the novel A_4_ location relative to the fixed A_1_ location object (Fig. 2C; top left; *F*_(2,43)_ = 1.53; *P* = 0.23). However, both chronically and acutely irradiated females exhibited impaired pattern separation and increased memory interference relative to control mice, where they were unable to demonstrate an appreciation for novelty of the A_4_ location object as compared to the object that had been in the A_2_ location during initial training (Fig. 2C; middle left; *F*_(2,43)_ = 6.36; *P* = 0.0038). Also compared to the control animals, the chronically irradiated female mice were more impaired in differentiation of the A_3_ location object from the update session relative to the novel A_4_ location (Fig. 2C; bottom left; *F*_(2,42)_ = 3.077; *P* = 0.057). Even though the chronically irradiated male mice showed impairment on the update session (day 4; Fig. 2B), during the test session (day 5; Fig. 2C; right) all male mice were able to similarly recognize the novelty of the object in location A_4_ relative to the objects in the fixed A_1_ (*F*_(2,43)_ = 0.45; *P* = 0.64) and initial A_2_ (*F*_(2,42)_ = 0.76; *P* = 0.47) locations, as well as the updated object in location A_3_ (*F*_(2,43)_ = 1.20; *P* = 0.31). These observations suggest that in our study GCR exposure induces impairments in hippocampal memory and pattern separation in female mice that do not ultimately manifest in the irradiated male mice.

**Fig. 3:**
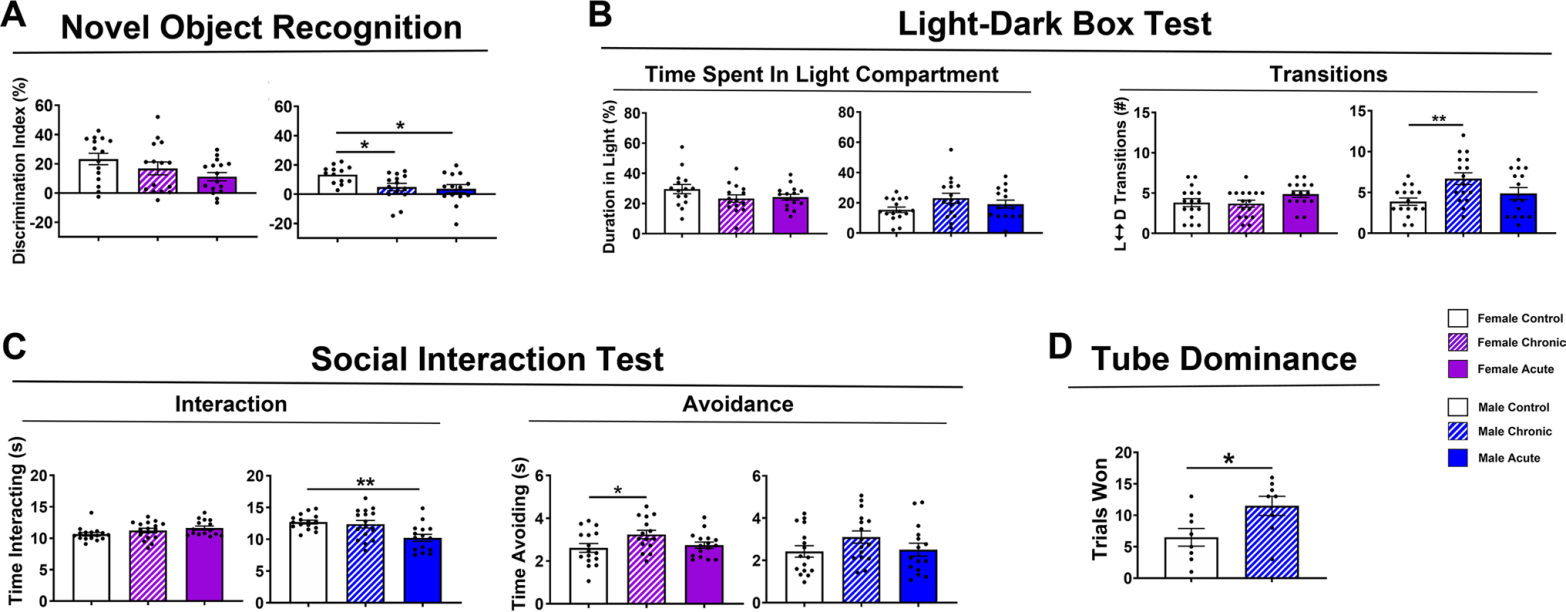
GCR Sim exposures induce memory impairments and changes in social behaviors. **A** Novel Object Recognition (NOR) testing indicated that chronically and acutely exposed males show significantly reduced discrimination index scores relative to controls, indicating no preference for the novel object. Acutely irradiated GCR females showed a trend toward reduced discrimination index scores compared to controls. **B** Using time spent in the light compartment of the light–dark box test (LDB) as a measure of anxiety-like behavior, none of the GCR-exposed animals were affected (left panel). Evaluation of numbers of transitions between the light and dark compartments on the LDB could suggest that chronic GCR males showed elevated number of transitions possibly suggesting increased frantic, anxiety-like behavior although analysis of time spent in each compartment do not support this conclusion (right panel). **C** During social interaction test (SIT), only acutely irradiated males showed reductions in social interactions with a novel mouse (left panel), while chronically irradiated females exhibited avoidance behavior (right panel). **D** Male mice exposed to chronic GCR Sim showed an increase in trials won compared with unirradiated control mice when tested on the Tube Dominance behavioral task, suggesting an increase in aggressive behavior. For NOR, LDB and SIT, data are the mean ± SEM (*N* = 13–16 mice/group); *P* values derived from one-way ANOVA followed by a Bonferroni’s multiple comparison test. For the Tube Dominance test, data are the mean ± SEM (*N* = 8 per group); *P* values derived from unpaired *t*-test. *P < 0.05, **P < 0.01

Following the OUL task, animals were tested sequentially on novel object recognition (NOR), the light–dark box (LDB) test and social interaction test (SIT). The NOR task depends on both the hippocampus and perirhinal cortex to test the animal’s ability to discriminate novelty [26]. In the NOR task, female mice exposed to acute GCR exhibited a trend toward a significantly impaired ability to discriminate the novel object compared to controls (Fig. 3A; left; one-way ANOVA: *F*_(2,43)_ = 2.67; *P* = 0.081), while chronic GCR females performed similarly to controls (*P* = 0.46). However, male mice exposed to either chronic or acute GCR Sim both exhibited significantly diminished novel object discrimination as compared to controls (Fig. 3A; right; one-way ANOVA: *F*_(2,38)_ = 4.67; *P* = 0.015).

Radiation exposures have also been found to alter mood [27, 28], with low doses of acute charged particle radiation causing increased anxiety-like behavior in mice [3, 7]. To investigate whether chronic or acute exposure to GCR Sim also triggers anxiety-like behavior, mice were administered the LDB test that is based on the tendency of anxious rodents to more actively avoid open brightly lit areas and exhibit reluctance to explore open environments. Such anxiety-like behaviors can manifest as reduced numbers of transitions between the dark and light compartments of the LDB testing arena or alternatively, as frantic darting behavior between the 2 compartments [29]. During LDB testing, irradiated female and male mice spent similar percent test times in the light compartment as their respective controls (Fig. 3B; left; female: one-way ANOVA: *F*_(2,42)_ = 1.81; *P* = 0.18; male: one-way ANOVA: *F*_(2,44)_ = 2.16; *P* = 0.13). Female GCR Sim-exposed mice also performed similarly to controls with regards to the number of light–dark compartment transitions made (Fig. 3B; right; one-way ANOVA: *F*_(2,44)_ = 2.06; *P* = 0.14). However, male mice exposed to chronic GCR Sim, but not acute GCR Sim, more frequently transitioned between the light and dark compartments compared to controls (Fig. 3B; one-way ANOVA: *F*_(2,44)_ = 5.09; *P* = 0.01) that could suggest increased anxiety-like behavior, although the lack of significant differences among groups for time spent in each chamber (Fig. 3B) and between male control and chronic GCR Sim-exposed mice on the OFT suggests otherwise (Supp. Figure 1B).

Next, we performed the SIT to examine social interaction behaviors that are found to depend on brain structures including the hippocampal and mPFC circuits [30]. Within a barrier-free arena each group-housed experimental mouse was allowed to interact with a novel mouse. The total time the experimental mouse spent interacting with the novel mouse or actively avoiding social interactions initiated by the novel mouse were recorded following established protocols [31]. Neither chronic nor acute GCR-exposed female mice showed impairments in the total amount of time spent in social interactions compared to control animals (Fig. 3C; one-way ANOVA: *F*_(2,44)_ = 2.41; *P* = 0.10). However, the female mice exposed to chronic GCR Sim, but not acute GCR Sim, spent significantly more time actively avoiding a novel mouse during the 10 min trials compared with unirradiated control mice (Fig. 3C; one-way ANOVA: *F*_(2,44)_ = 3.21; *P* = 0.050). Conversely, male mice exposed to acute GCR Sim, but not chronic GCR Sim, spent significantly less time interacting with a novel mouse, compared to controls (Fig. 3C; one-way ANOVA: *F*_(2,44)_ = 7.21; *P* = 0.0020). No group differences were observed when comparing active avoidance behavior among the irradiated male cohorts (Fig. 3C; one-way ANOVA: *F*_(2,44)_ = 1.67; *P* = 0.20). These data suggest that while exposure to GCR Sim alters some aspects of social interaction behaviors in both female and male mice the exact nature of the impact varies among the different groups. Lastly, we observed no GCR Sim-induced changes in associative fear memory in either male or female mice (Supp. Figure 2).

In a separate cohort of male mice, we examined social hierarchy or aggression using the tube dominance test, where a novel mouse and an experimental mouse are placed facing each other at opposite ends of a narrow tube and meet in the middle. This test was performed at ~ 4.5 months after irradiations were completed. The mouse that forces its opponent out of its way is designated the ‘winner’ and more socially dominant [32]. The mouse that retreats from the tube is designated the ‘loser’ and shows traits of being more subordinate. In the tube dominance test, male mice exposed to chronic GCR Sim were observed to achieve substantially more wins compared to control animals, indicating radiation-induced elevations in dominant or aggressive behavior. (Fig. 3D; *t*-test: *t*_(14)_ = 2.41; *P* = 0.031). Further studies in the same cohort of male mice showed no additional deficits in spatial memory or general locomotion (Fig. S1).

### Hippocampal synaptic plasticity is diminished following GCR Sim exposure

Synaptic plasticity mechanisms effectuate activity-dependent dynamic rebalancing within the intricately interconnected hippocampal network of excitatory neurons and diverse GABAergic interneurons. Repeated activation of synaptic inputs from CA3 to CA1 pyramidal neurons through high frequency Schaffer collateral stimulation has long been known to produce prolonged enhancement of synaptic activity, known as long-term potentiation (LTP), thought to represent a cellular basis of memory [33]. Given the observed deficits in cognitive processes involving hippocampal and cortical networks in mice exposed to either acute or chronic GCR Sim, we followed up our behavioral testing by determining whether hippocampal LTP became perturbed in GCR Sim-exposed mice at 5 months after irradiations.

**Fig. 4:**
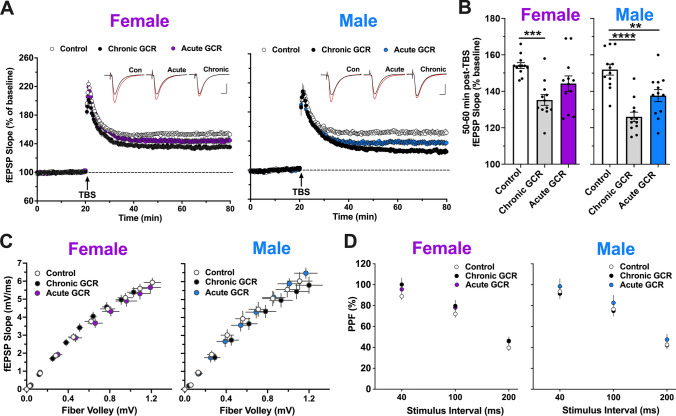
Hippocampal long-term synaptic plasticity is perturbed by GCR Sim exposure. Extracellular field recordings of CA1 dorsal hippocampus apical dendrite responses to Schaffer collateral stimulation at 5 months following completion of chronic and acute GCR Sim exposures. **A** Following a stable 20 min baseline recording, a single train of theta burst stimulation (TBS; arrow) was applied, and then, recordings were continued for an additional 60 min. The time course shows that TBS-induced long-term potentiation (LTP) was markedly reduced in slices from chronically irradiated female and both groups of GCR Sim-exposed male mice compared with slices from respective control animals. Representative traces collected during baseline (inset; black line) and 60 min post-TBS (red line). Scale bars indicate 0.4 mV/5 ms. **B** Chronically GCR Sim-exposed female mice showed a marked reduction in LTP at 60 min post-TBS relative to control mice (left). Field excitatory postsynaptic potential (fEPSP) slope was significantly reduced 60 min post-TBS in slices from both chronically and acutely GCR-exposed male mice (right). **C** The relationships between stimulation current and fEPSP slope were not detectably different between groups. **D** Transmitter release kinetics, as assessed with paired pulse facilitation (PPF), were also comparable among all animals. Data are mean ± SEM (total *N* = 6 mice per group; 1 slice/hemisphere per mouse); *P* values for mean potentiation and fEPSP slope derived from one-way ANOVA followed by a Bonferroni’s multiple comparison test. *P* values for PPF derived from two-way ANOVA. **P < 0.01, ***P < 0.001. ****P < 0.0001

The delivery of theta burst stimulation (TBS) to the Schaffer collateral produced a robust and immediate increase in LTP, measured as the relative change in the slope of evoked field excitatory postsynaptic potentials (fEPSPs) generated by CA1 apical dendrites (Fig. 4A). fEPSP slope then gradually decayed to a stable level of potentiation in brain slices from all treatment groups of female and male mice. LTP levels in these hippocampal slices were found to be consistent with our prior reports [4, 8]. The level of potentiation in fEPSP slope maintained 50–60 min post-TBS was significantly reduced in the hippocampus of the chronically, but not acutely, GCR Sim-exposed female mice (Fig. 4B; left; one-way ANOVA: *F*_(2,33)_ = 9.26; *P* = 0.00060; Bonferroni post hoc: *P* = 0.00030, *P* = 0.062, respectively) as compared to controls. Male mice exhibited reduced mean potentiation in both chronically and acutely GCR Sim-exposed mice (Fig. 4B; right; one-way ANOVA: *F*_(2,33)_ = 18.85; *P* < 0.0001; Bonferroni post hoc: *P* < 0.0001, *P* = 0.0039, respectively) relative to controls. These findings support the hypothesis that exposure to simulated GCR adversely impacts network mechanisms of synaptic plasticity that underlie critical learning and memory processes.

Measures of baseline synaptic transmission were found to be unaltered in either chronic or acute GCR Sim-exposed mice relative to control animals. Specifically, the slope of the input/output curves for evoking fEPSPs in chronic and acute GCR Sim hippocampi were no different from in control mice (Fig. 4C; female: one-way ANOVA: *F*_(2,27)_ = 0.051; *P* = 0.95; males: one-way ANOVA: *F*_(2,27)_ = 0.010; *P* = 0.99). Accordingly, there were no significant changes between treatment groups in the presynaptic plasticity of transmitter release, as measured in a paired pulse facilitation assay (Fig. 4D; female: two-way ANOVA: *F*_(2,33)_ = 1.014; *P* = 0.37; males: one-way ANOVA: *F*_(2,33)_ = 0.50; *P* = 0.61). Together, these results show that GCR Sim exposure impairs LTP in the hippocampus.

### GCR Sim exposure elicits limited alterations in a CA1 synaptic signaling

As GCR Sim irradiation disrupts both hippocampus-related behaviors and hippocampal network plasticity mechanisms, we next investigated the manner in which accurately modeled space radiation exposures may have perturbed the electrophysiological properties of individual neurons. Previously, we and others have observed that acute single-ion [7, 34, 35] or multiple-ion charged particle irradiation [3], as well as chronic neutron irradiation [8], is capable of disrupting hippocampal neuron signaling. Therefore, we sought to determine whether irradiation with acute or chronic GCR Sim paradigms that more accurately recapitulate the space radiation environment likewise altered the electrophysiological properties of hippocampal neurons.

**Fig. 5:**
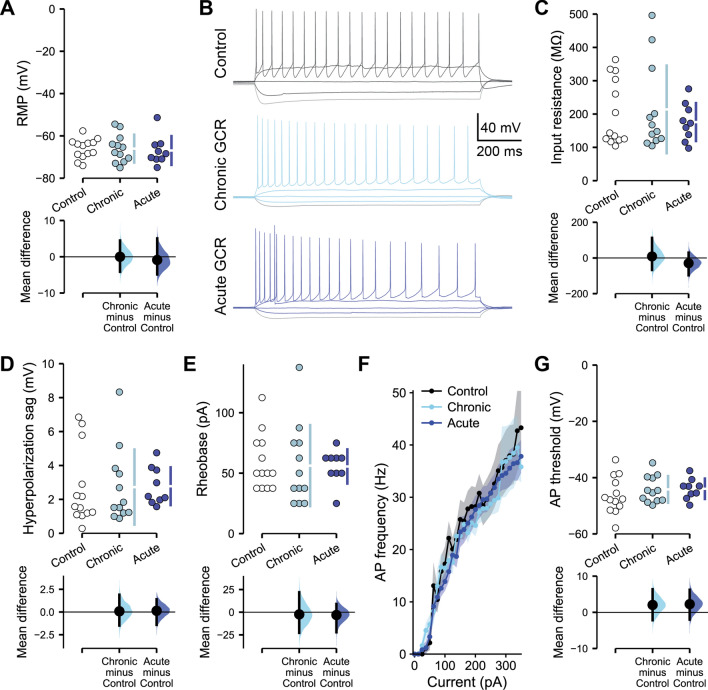
GCR Sim exposure does not alter the intrinsic electrophysiological properties of CA1 pyramidal neurons in male mice. All data are from whole cell current clamp recordings of CA1 pyramidal neurons from the superficial layer of the dorsal hippocampus, 2–4 months following either chronic or acute exposures of male mice to GCR Sim. **A** Resting membrane potential (RMP) was unchanged between groups. **B** Representative examples of responses to a range of brief current injections in control and irradiated neurons. There was no alteration in the input resistance (**C**), sag during a − 100 pA hyperpolarizing current injection (**D**) or rheobase current required to evoke an action potential (**E**) between groups. **F** Action potential (AP) frequency remained equivalent across a range of current injections and **G** the threshold potential for action potential initiation remained unchanged. Data are Control: 5 animals, 13 cells; Chronic: 5 animals, 12 cells; acute: 4 animals, 9 cells. **A**, **C–E**, **G** Cumming estimation plots show raw data on the top axis and a bootstrapped sampling distribution on the bottom axis; black dots depict the mean difference between groups and the 95% confidence interval is indicated by the ends of the vertical black bars. **F** Data are mean ± SEM. *P* values derived from linear mixed-effect model regression or two-way ANOVA

We initially assessed whether either chronic or acute GCR Sim exposures produced changes in the intrinsic electrophysiological properties of hippocampal pyramidal neurons within the CA1 superficial layer at 2–4 months post-irradiation of the male mice (Fig. 5). Neither GCR Sim irradiation paradigm altered the resting membrane potential (RMP) of pyramidal neurons (*F*_(2,8.17)_ = 0.050, *P* = 0.95, linear mixed-effect modeling (LMM) (Fig. 5A). Assessing neuronal responses to a range of brief current injections allowed for measurement of other cell-intrinsic properties (Fig. 5B). We observed no radiation-induced changes in either the input resistance (Fig. 5C; *F*_(2,31)_ = 0.37, *P* = 0.70, LMM) or hyperpolarization sag amplitude responses to -100 pA current injections (Fig. 5D; *F*_(2,8.87)_ = 0.0020, *P* = 0.10, LMM) of CA1 pyramidal neurons. Measuring neuronal excitability based on how readily action potential (AP) firing could be evoked, GCR Sim exposure was found to alter neither the rheobase current required to first evoke an AP (Fig. 5E; *F*_(2,31)_ = 0.050, *P* = 0.96, LMM) nor AP firing frequency across a range of current injections (Fig. 5F; *F*_(2,859)_ = 2.19, *P* = 0.11, two-way ANOVA). There was also no clear impact of GCR Sim exposure on the characteristics of individual APs, such as the threshold potential for AP initiation (Fig. 5G; *F*_(2,7.24)_ = 0.66, *P* = 0.55, LMM). Additional unaltered intrinsic electrophysiological properties and statistical parameters for the above measurements are included in Supplemental Table 1. Overall, we did not observe any changes to the intrinsic properties of CA1 pyramidal neurons following either chronic or acute GCR Sim exposures of male mice.

**Fig. 6:**
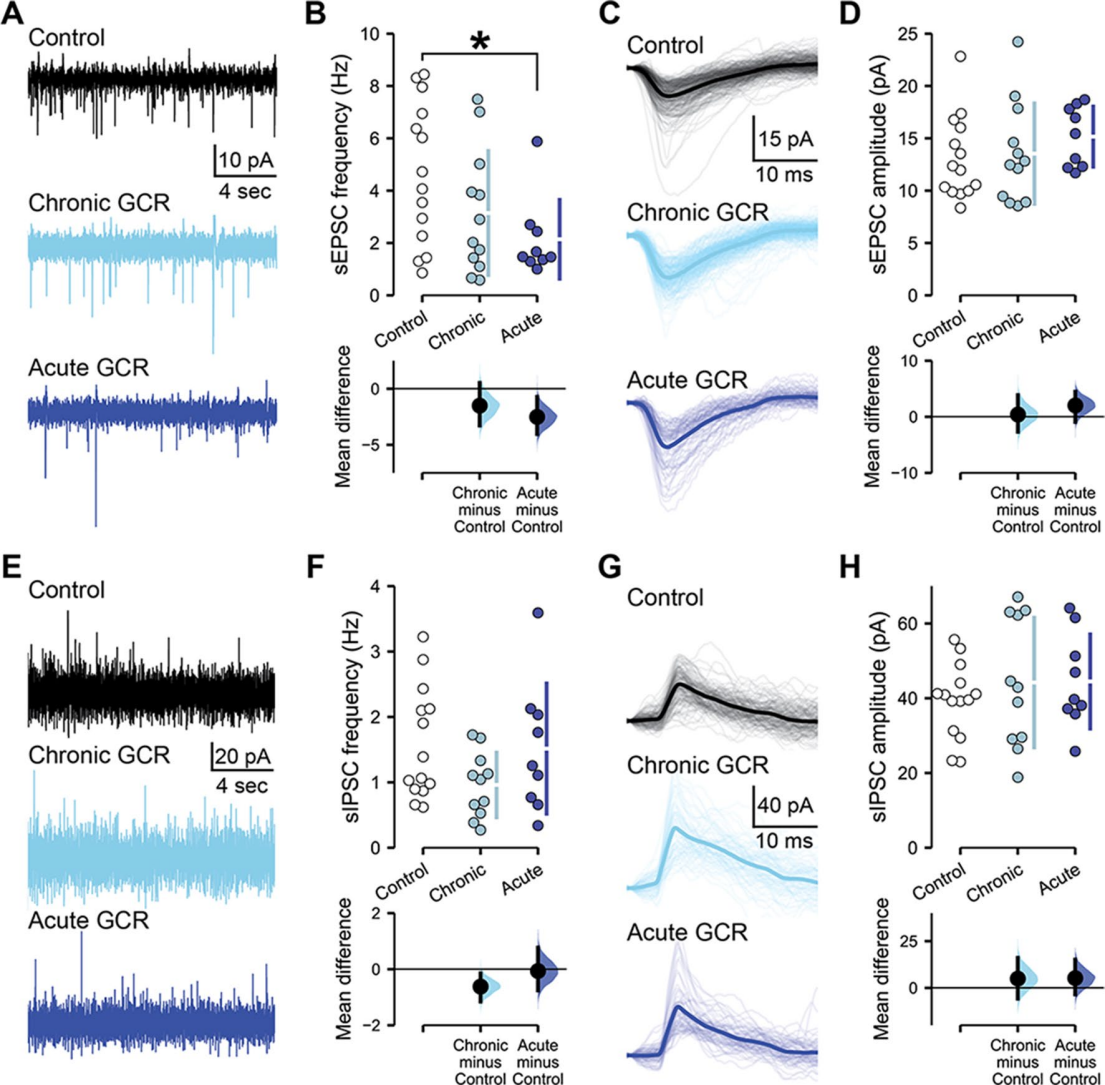
Acute exposure to GCR Sim preferentially suppresses excitatory synaptic signaling in male mice. All data are from whole cell voltage clamp recordings of CA1 pyramidal neurons from the superficial layer of the dorsal hippocampus, 2–4 months following either chronic or acute irradiations of male mice. **A** Representative examples of spontaneous excitatory postsynaptic current (sEPSC) recordings from control and GCR Sim-exposed neurons. **B** The frequency of sEPSCs was reduced in male mice following acute irradiation. **C** Aligned examples of sEPSCs in representative control and irradiated neurons. Light lines show individual sEPSCs, while darker lines display the average sEPSC during a 200 s recording from that neuron. **D** sEPSC amplitude remained similar between groups. **E** Representative examples of spontaneous inhibitory postsynaptic current (sIPSC) recordings from control and GCR Sim-exposed neurons. **F** Frequency and **G, H** amplitude of sIPSCs was equivalent between groups. Data are Control: 5 animals, 14 cells; Chronic: 5 animals, 12 cells (11 cells for sIPSCs); Acute: 4 animals, 9 cells. **B, D, F, G** Cumming estimation plots show raw data on the top axis and a bootstrapped sampling distribution on the bottom axis; black dots depict the mean difference between groups and the 95% confidence interval is indicated by the ends of the vertical black bars. *P* values derived from linear mixed-effect model regression. *P < 0.05

While neither acute nor chronic GCR Sim exposures disrupted the intrinsic properties of CA1 pyramidal neurons, we have previously observed changes in hippocampal synaptic connectivity following single-ion GCR, multiple-ion GCR and chronic neutron irradiation paradigms [3, 7, 8, 17, 35]. Furthermore, low doses of single-ion GCR are known to disrupt dendritic spine morphology within the hippocampus [5, 6, 36]. Thus, we next performed electrophysiological recordings of the spontaneous excitatory and inhibitory postsynaptic activity received by CA1 pyramidal neurons, to examine any changes in response to either chronic or acute GCR Sim exposures in male mice (Fig. 6). Recording the spontaneous excitatory postsynaptic currents (sEPSC) received by CA1 pyramidal neurons, we found that irradiation altered sEPSC frequency (Fig. 6A, B; *F*_(2,32)_ = 3.37, *P* = 0.047). Relative to control mice, we observed a large effect size decrease in sEPSC frequency after acute GCR Sim exposure (mean difference (*M*_diff_) = − 2.52 Hz, 95% CI [− 4.04, − 0.72]; *d* = − 1.08, 95% CI [− 1.91, − 0.17], *P* = 0.044) that was not present following chronic GCR Sim exposure (*M*_diff_ = − 1.51 Hz, 95% CI [− 3.28, 0.51]; *d* = − 0.59, 95% CI [− 1.41, 0.29], *P* = 0.243). Although sEPSC frequency was altered by GCR Sim, the amplitude of the sEPSCs received by CA1 pyramidal neurons was not changed (Fig. 6C, D; *F*_(2,9.36)_ = 0.10, *P* = 0.91). Likewise, spontaneous inhibitory postsynaptic current (sIPSC) frequencies (Fig. 6E, F; *F*_(2,6.35)_ = 1.50, *P* = 0.29) and amplitudes (Fig. 6G, H; *F*_(2,11.17)_ = 0.49, *P* = 0.62) remained similar to control levels after acute or chronic GCR Sim irradiation. Other unaltered measurements of synaptic signaling properties and statistical parameters for these endpoints are included in Supplemental Table 2. Overall, we could identify no changes in the electrophysiological properties of CA1 pyramidal neurons in response to chronic GCR Sim exposures in male mice, whereas acute irradiation appears more capable of disrupting hippocampal excitatory inputs.

### GCR Sim exposure resulted in alterations in large synapse complexity

**Fig. 7:**
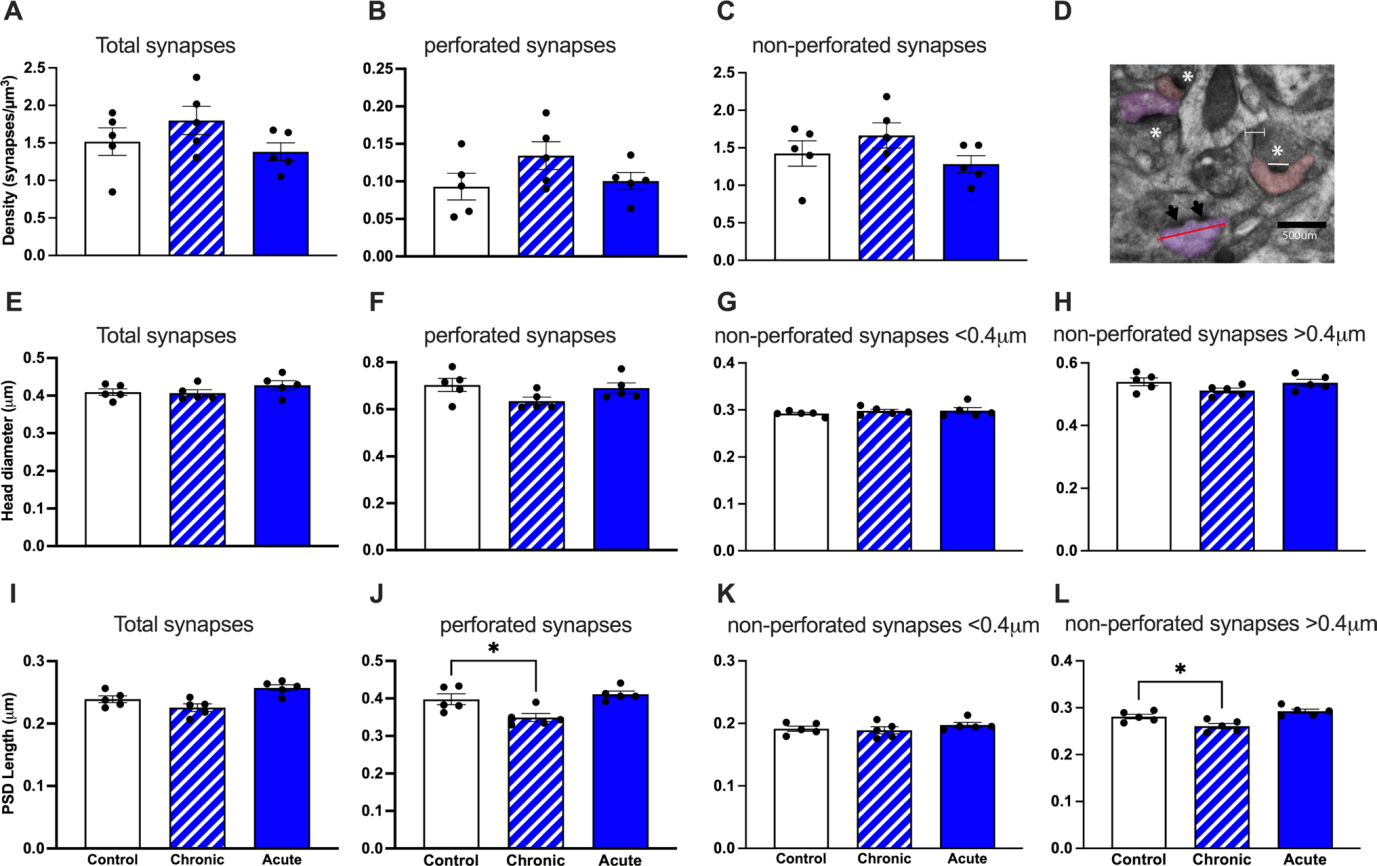
GCR Sim exposure alters PSD length in large, > 0.4 mm diameter synapses in CA1 pyramidal neurons of male mice. There were no significant changes in total synapse density, perforated synapse density or non-perforated synapse density compared to controls. **A** total synapse density, **B** perforated synapse density and **C** non-perforated synapse density. **D** Representative electron micrograph depicting non-perforated synapses (white asterisks), perforated synapses (arrow heads) and measurements of PSD length (white line) and head diameter (red line). Scale bar = 500 nm. **E–H** HD measurements for all synapses, perforated synapses, non-perforated synapses < 0.4 mm and non-perforated synapses > 0.4 mm, respectively. **I** PSD length in all synapses showed smaller PSDs in chronic GCR mice. **J** Perforated synapse PSD lengths were significantly reduced following chronic GCR exposure. **K** PSD lengths in non-perforated synapses < 0.4 mm in HD. **L** Non-perforated synapses > 0.4 mm in HD were significantly reduces in chronically exposed GCR mice. *N* = 5 mice per group. Data are mean ± SEM, one-way ANOVA *P < 0.05, **P < 0.01

Given the effect of GCR exposure on behavior, hippocampal plasticity and electrophysiology, we examined synapse density and morphology in the stratum radiatum of the CA1 region of the hippocampus as well as layer II/III of the medial prefrontal cortex (mPFC) of male mice at the ultrastructural level using quantitative EM (Supp. Figure 3). To answer the question of whether changes in the different types of synapses may contribute to in the behavioral and electrophysiological changes observed, we differentiated all synapses into either perforated or non-perforated synapses and assessed postsynaptic density (PSD) length, and head diameter (Fig. 7). Perforated and non-perforated synapses constitute separate synaptic populations from early in development [37]. Non-perforated or simple synapses have a unique and continuous PSD. Perforated synapses are morphologically characterized by a discontinuity in the PSD (Fig. 7D, black arrows). They are found on larger spines and are stable synapses implicated in memory-related plasticity [38]. They are also proposed to be a structural correlate of enhanced synaptic efficacy as spines with perforated synapses have increased synaptic strength due to the membrane expansion, insertion of new receptors into the two PSDs and in the synaptic membrane and the creation of two independent release sites, with their own release probabilities [39–41]. We did not observe any significant group differences in total, perforated or non-perforated synapse density (Fig. 7A–C). Analysis of total synapses and perforated synapses revealed no significant change in HD between irradiated mice and controls (Fig. 7E, F). Our previous studies have classified mouse spines with head diameters < 0.4 μm as smaller, thin spines and > 0.4 μm as larger, mushroom spines [16, 17]. When we apply this parameter to non-perforated synapses, we found no differences in HD between GCR-exposed mice and controls (Fig. 7G, H). Analysis of PSD length showed a significant decrease in chronic GCR-exposed mice (Fig. 7I; *F*_(2,12)_ = 8.41; *P* = 0.005). Further analyses of perforated synapse PSD length also found significant differences (Fig. 7J; *F*_(2,12)_ = 11.75; *P* = 0.001) and pairwise comparisons showed significant differences between control vs. chronic conditions (*t*_(12)_ = 3.51; *P* = 0.013). Once the data for non-perforated synapses was separated based on HD < 0.4 μm and > 0.4 μm, we found significant results in < 0.4 μm HD synapses (Fig. 7L; *F*_(2,12)_ = 3.92; *P* = 0.049). Interestingly, examination of PSD lengths on non-perforated synapses with > 0.4 μm HD showed significant effects of chronic GCR exposure (Fig. 7K; *F*_(2,12)_ = 10.27; *P* = 0.002) with the pairwise comparison showing significant differences in control vs. chronic (*t*_(12)_ = 2.86; *P* = 0.043). These data indicate that larger, mushroom-like spines, have smaller PSD lengths as a result of chronic GCR sim exposure.

### GCR Sim exposure results in alterations in myelination in the corpus callosum

**Fig. 8:**
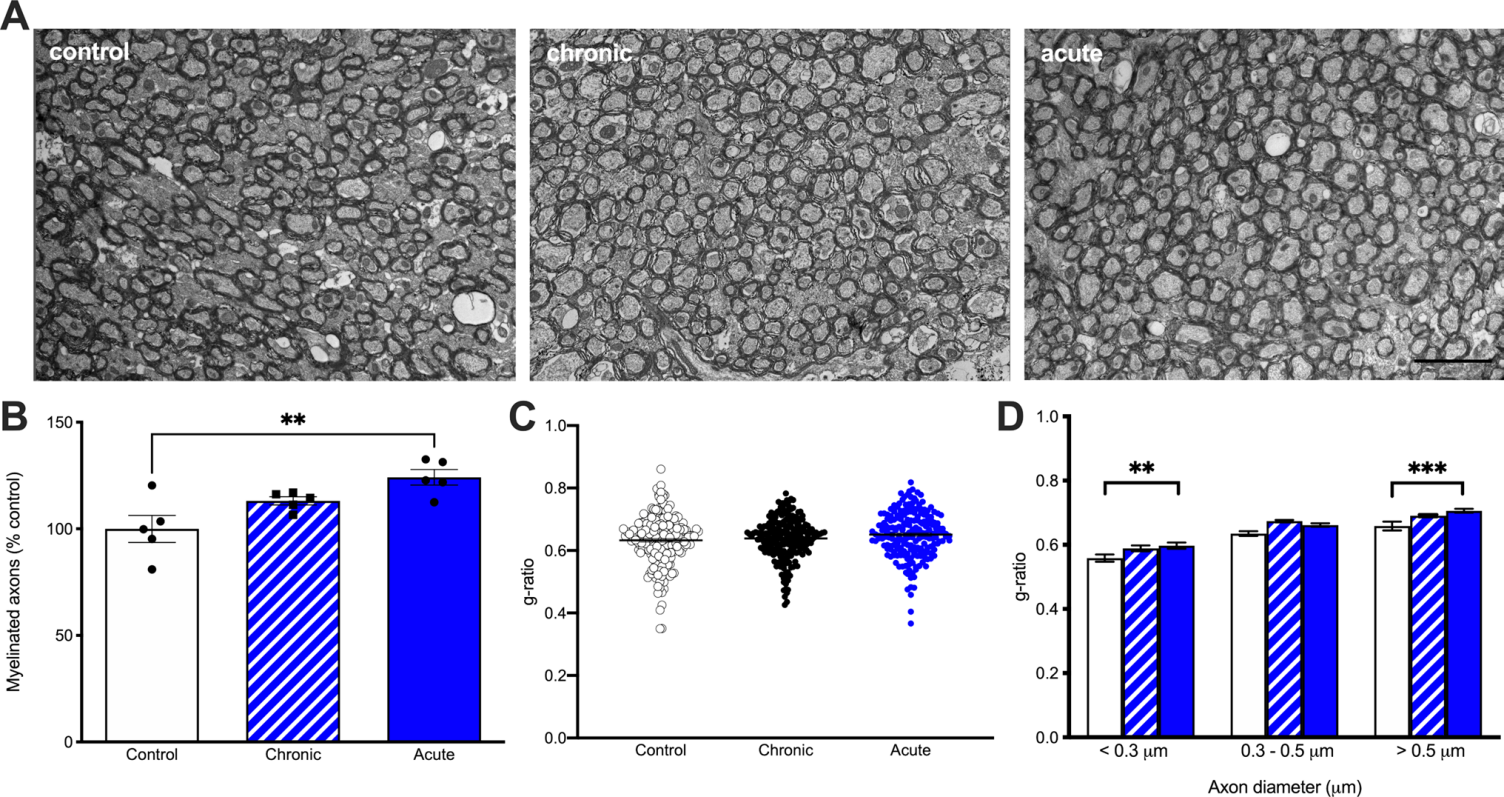
Acute exposure to GCR Sim results in myelin degeneration in male mice. **A** Representative images from the corpus callosum from control (*N* = 5 mice; *N* = 4303 axons), chronic (*N* = 5 mice; *N* = 4868 axons) and acute (*N* = 5 mice; *N* = 5343 axons) GCR-exposed mice. Scale bar = 2 mm. **B** There was an increase in the percent of myelinated axons following acute irradiation with no differences between chronic irradiation and controls. **C** There is no significant difference in overall g-ratios in chronic or acute irradiated mice compared to controls. **D** Acute irradiation results in less myelin in the smallest (< 0.3 mm) and largest axons (> 0.5 mm). *N* = 5 mice per group. *P* values derived from linear mixed-effect model regression. *P < 0.05

We next asked whether acute and chronic irradiation altered myelination (Fig. 8A). We quantified the percentage of myelinated axons as well as the g-ratio of axons (the ratio of inner to outer diameter of the myelinated axon) providing a morphometric analysis of the axons. Acute GCR Sim irradiation resulted in a significant increase in the percent of myelinated axons compared to controls (Fig. 8B; *F*_(2,12)_ = 7.67; *P* = 0.007 one-way ANOVA). There was no difference between chronic exposure and controls. Comparison of total g-ratios also did not show any differences between all groups of mice (Fig. 8C). When we separated axons by size, we found that the smaller caliber axons, < 0.3 μm in diameter, and large calibur axons, > 0.5 μm in diameter, showed an increase in *g*-ratio indicative of thinner myelin sheaths in acute irradiated mice (Fig. 8D, F_(4,~617.14)_ = 2.80, *P* = 0.026; < 0.3 μm axons control vs acute *t*_(~93.5)_ = − 2.23; *P* = 0.029 and for the large bin, *t*_(~103.9)_ = − 2.64; *P* = 0.009, LMM). There was no difference between chronically irradiated mice and controls.

## Discussion

As NASA and others move forward with plans for missions to the Moon and then Mars, it is of critical importance that the potential health risks associated with deep space radiation exposures are well understood. A large body of literature has clearly established that single-ion charged particle exposures elicit significant CNS impairments [1, 42]. Recent studies of simplified GCR Sim exposures using 5–6 beams including protons, ^28^Si, ^4^He, ^16^O, ^56^Fe have moved in the direction of more realistically modeling the space radiation environment and the subsequent detrimental CNS responses [3, 4, 9]. The general consensus of these CNS studies, using the best representative simulations of space radiations that are available, indicate that neurocognitive complications starting at approximately one month post-exposure and do not resolve. Further, these studies suggest that the functional changes in cognition do not track with the microdosimetric properties of the absorbed doses for doses at least ≤ 50 cGy. While these studies are informative, they still fall short of accurately representing the complex mixture of ions and energies of radiation to which an astronaut would be exposed during long-duration spaceflight, such as a Mars mission. The complex GCR Sim paradigm developed by the NASA Space Radiation Laboratory includes 33 distinct charged particle beams, predominated by protons and ^4^He particles of various energies interspersed with infrequent beams of representative heavy ions [10]. Past single-ion studies have provided important insights into the biological risks of certain ion exposures; however, the 33-beam acute GCR Sim exposure captures more representative CNS hazards that arise when exposed to space-relevant radiation fields. While an acute 33-beam GCR Sim paradigm has been utilized by Kiffer and colleagues to evaluate behavioral changes in male mice [11], our study includes both sexes and a detailed quantification of synaptic plasticity and structure. Furthermore, our chronic GCR Sim exposure paradigm represents the most realistic ground-based model available to date. As such, our study advances our understanding of how the CNS responds to a complex mixture of energetic charged particles, by investigating effects across a range of cellular and neural networks and behavioral functions.

To determine how the GCR Sim exposures affect the CNS and to link animal behavior to neuronal networks previous displaying sensitivity to other space radiation models, we employed cognitive testing paradigms that primarily interrogate hippocampus–medial prefrontal cortex network functions. To determine how GCR Sim exposures impact long-term hippocampus-dependent memories and memory updating in female and male mice, we used the OUL testing paradigm in addition to novel object recognition and fear extinction testing. The OUL task is designed to be more sensitive than a novel place recognition assay [25]. Interrogating hippocampal function with the OUL task can simultaneously assess multiple memory traces, thereby having stronger cross-species correlates with humans than more simplified rodent behavior testing paradigms [42]. OUL starts with novel place recognition but includes an additional object relocation phase that elevates task rigor to determine how overlapping associative memories can be segregated. Therefore, a key strength is that the OUL task discriminates between updates or interference to existing memories, rather than the de novo formation of new associations. As such, the OUL assay incorporates two overlapping events that require dentate gyrus-dependent pattern separation [43] and tests whether new learning will occur despite this prior interference. Our previous studies of male mice exposed to simplified GCR Sim demonstrated normal memory acquisition but significant impairments in memory updating [4]. While the chronically irradiated male mice performed poorly during their update session, they and the acutely exposed male mice were successful during the test day, accessing original memories and performing at control levels. Conversely, the female mice exposed to chronic or acute GCR Sim had intact original memories, but failed to update the learned information regarding the most novel object location presented on the final testing day, suggesting that GCR exposure induces impairments in hippocampal memory reconsolidation and cognitive reserves in female mice that do not manifest to the same extent in the irradiated male cohorts.

While the neural mechanisms that facilitate reconsolidation-based updating to modify memories have been investigated extensively [25, 44], the circuits controlling these behaviors and how complex irradiation paradigms impact these circuits are far less understood. This confounds interpretation of present results obtained in other behavioral paradigms, as changes in the NOR, LDB and SIT testing paradigms manifested differently between acute and chronic exposures and between the sexes, pointing to the nuances of space radiation exposure on CNS functionality. The differential dependence of cortical and hippocampal circuitry to radiation-induced change and their sensitivity to inflammatory and hormone modulation may explain this equivocality between the sexes regarding their performance on particular testing paradigms. Past work has shown a resistance of female mice to space radiation-induced cognitive decline [45, 46], while other work has not [47]. The current study did not evaluate the level of sex steroids in either male or female mice before or after the completion of radiation exposures, but sex differences and the role of sex hormones are an area clearly requiring additional study. Interestingly, male mice were found to exhibit increased aggressive-like behavior, which did not translate to changes in associative fear memory, the latter of which was used to evaluate changes in cognitive flexibility using a fear extinction task. Typically, fear memories are robust and persistent [48], involving rapid, strong associations, but due to their aversive nature can confound simultaneous access to original and updated memories [49]. Thus, it is noteworthy that female and male cohorts exposed to either of the two GCR Sim paradigms exhibited no impairments on the fear extinction memory test. Our past studies have shown significant impairments in this task which involves intact hippocampal function [8] in concert with the medial prefrontal cortex and amygdala [48]. While the reasons for this remain uncertain, the impact of GCR exposure on prelimbic and infralimbic circuitry of the mPFC that regulate fear expression and suppression, respectively, may be offset, exhibiting relative equal sensitivity to radiation-induced change.

Memory impairments, such as we observe following both chronic and acute GCR Sim exposures are often associated with underlying disruptions of synaptic plasticity processes, including LTP [33, 50]. Indeed, impaired LTP is observed in the hippocampus following several irradiation paradigms, including single-ion [51, 52], 5-ion [4], chronic neutron [8] and our current GCR Sim results. While we observe behavioral deficits and impaired LTP persisting months following GCR Sim irradiation, compensatory mechanisms may eventually allow the brain to mollify radiation-induced damage. Indeed, following an acute 100 cGy ^56^Fe irradiation mice initially show spatial memory and LTP deficits, yet by 6 months after exposure both traits show enhancements that remain long term [52]. Homeostatic plasticity mechanisms enable neuronal networks to cope with insults by regulating other cellular properties, such as ion channel expression, into alternative states that help stabilize overall activity. However, while plasticity mechanisms allow a network to tolerate disruptions, they may leave the network in a destabilized state that is more vulnerable to cognitive impairment and epilepsy following subsequent stresses [53, 54].

In past experiments modeling irradiation with single particle types, including protons [34], ^4^He [7] or chronic neutrons [8], we have generally observed changes in neuronal intrinsic properties that indicate a reduction in network excitability. However, as with both acute and chronic GCR Sim exposures, we do not detect any persistent changes in hippocampal intrinsic excitability following irradiation with a mixed beam of 5 ions [3]. The exact reasons why mixed radiation fields appear to produce fewer apparent changes in the functional properties of individual neurons is unclear but may be due to counteracting and/or compensatory mechanisms responding to different ion energies and linear energy transfer. Elucidating these types of microdosimetric and/or compensatory responses was not the goal of the present study, where we focused on critical functional and neurobiological outcomes to GCR Sim exposures.

While GCR Sim exposures did not change the neuronal intrinsic characteristics we assessed, we do observe that acute GCR Sim exposures disrupt synaptic signaling properties. Similar reductions in the net proportion of excitatory synaptic signaling received by neurons following acute particle irradiation include suppressed EPSC frequency following ^4^He exposures [55] and increased amplitudes of IPSCs after proton [7] and 5-ion irradiation [3]. Such changes are in line with how acute charged particle exposures are known to damage structural elements, such as dendrites and dendritic spines, that are necessary for proper synaptic signaling [5, 7, 36]. What is less clear is why chronic GCR Sim exposures, which include positively charged protons, appear less likely to alter synaptic signaling properties. Although chronic neutron irradiation suppressed EPSC frequencies in CA1 neurons [8], no similar measurements have been performed following chronic charged particle exposures. Additionally, analysis of PSD indicated decreased length, particularly for mushroom spines, that might contribute to reduced synaptic efficiency. Similarly, while not conclusive or robust, changes in myelination thickness relative to axon size could indicate compromised axonal integrity [16]. Future investigations into the impacts of chronic particle irradiation on dendrites, dendritic spines and synaptic structures may help resolve these uncertainties.

The lack of apparent radiation-induced alterations in functional neuronal properties of CA1 pyramidal neurons, outside of reduced sEPSC frequency following acute GCR Sim exposures, does not rule out that other neuronal populations are being more substantially disrupted. We have previously observed that the functional properties of other cell populations such as hippocampal cannabinoid type 1 receptor-expressing basket cells [56] and perirhinal cortex regular spiking principal cells [7] are altered by charged particle irradiation. There is also evidence that several other brain regions, including the hypothalamus, striatum and nucleus accumbens, are sensitive to GCR exposures [56]. Even within the hippocampus, radiation exposures are known to alter adult neurogenesis of hippocampal neurons [52, 57]. However, these findings are mixed. Sahay and colleagues found that integration of newborn neurons was important for cognitive processes [58], but Whoolery et al., found that single-ion space radiation exposures improved pattern separation in a dentate gyrus-dependent touch screen task of male mice despite impaired neurogenesis [59]. Given the age of animals and doses used in our study, the overall impact of neurogenesis is questionable within this context, and not readily amenable to whole cell electrophysiology assays.

Overall, our study is the first to examine how high-fidelity simulations of space-relevant radiation exposures reveal risks that astronauts might encounter at multiple levels of central nervous system function. Until well-defined cohorts of animals are exposed to the deep space environment and space flight stressors that would be expected on a Mars mission, the ground-based studies by us and others remain the gold standard for assessing the impact of space-relevant radiation exposure on the CNS. Clearly, future studies will be required to further elucidate sex differences in the space radiation response of the CNS, as will the combined effect of irradiation with other mission-relevant stressors such as sleep disruption. While both acute and chronic GCR Sim exposures disrupt critical cognitive processes and underlying neuronal network plasticity, alteration to the functional properties of individual neurons appear to be less likely at more mission-relevant doses and dose rates. Given that the mechanisms underlying the persistent effects of space radiation exposures to brain function remain elusive, the hazards to astronaut CNS capabilities are unclear. Given current data, though, those risks may in fact be less than predicted by earlier studies evaluating less refined irradiation paradigms. However, clear evidence of cognitive deficits arising in both male and female animals regardless of the time course of GCR Sim exposure indicate that radiation risks need to be carefully considered when planning future human exploration of the Moon and Mars**.**

## Supplementary Information

Below is the link to the electronic supplementary material.

Supplementary file1 (DOCX 171 KB)

Supplementary file2 (TIF 1385 KB)

Supplementary file3 (TIFF 1894 KB)

Supplementary file4 (TIFF 1523 KB)

## Data Availability

Most of the data generated and analyzed are contained in this published article and its supplementary information files or are available from the corresponding author on reasonable request.
