# Supplementary material for: Galactic cosmic radiation exposure causes multifaceted neurocognitive impairments
Source: Cell Mol Life Sci. 2023 Jan 6;80(1):29. doi: 10.1007/s00018-022-04666-8 (PMC9823026; doi:10.1007/s00018-022-04666-8)
Supplement: Supplementary file 1 — Supplementary file1 (DOCX 171 KB) [file 18_2022_4666_MOESM1_ESM.docx]

**Supplemental Information**

**Methods**

**Animals and irradiations**

During whole-body irradiations, sets of 2-3 cage mate mice were loosely constrained together in Lucite containers lined with absorbent padding and with well-ventilated breathing holes. Each day, the same mice from each home cage were grouped together and placed into the same container. Any day that the chronically irradiated mice were restrained and irradiated, all other mice were also similarly restrained in their designated boxes within the animal holding room. For each exposure or sham exposure day, the time between when the mice were loaded into their boxes, irradiated, and then subsequently unloaded back into their respective home cage generally averaged no more than 2 hours. Within 5 days of completion of the GCR Sim exposures mice were shipped to their respective institutions directly from the NASA Space Radiation Laboratory (NSRL) at Brookhaven National Laboratory (*i.e.,* Harvard, UC Irvine, Stanford; **Fig. 1**).

**Cognitive testing**

The Object in Updated Location (OUL), Novel Object Recognition (NOR), and Social Interaction Test (SIT) assays were conducted and recorded as described previously (1). All behavioral testing was conducted beginning ~2 months after the conclusion of GCR exposures and completed within 3 months of testing (*i.e.* 5 months post-irradiation).

***Objects in Updated Locations (OUL) Task***

The OUL task was conducted following our previously published protocol starting approximately 2 months after completion of the irradiations (**Fig. 2**). (1). Briefly, mice were handled for 4 days followed by 6 consecutive days of habituation to the testing arena. Mice were trained with 2 identical objects in specific locations (A_1_ and A_2_; fixed and initial locations, respectively) for 3 days in the habituated context (10 minutes) where the mouse was allowed 5 minutes to explore the objects. Twenty-four hours later, mice were given a 5 minute update session in which the A_2_ object was moved to location, A_3_. Finally, mice were given a 5 minute retention test in which mice were exposed to the 3 objects in previously experienced A_1_, A_2_, A_3_ locations and a fourth identical object in a novel A_4_ location. Memory for the update was determined via comparison of novel A_4_ location exploration to A_3_ location exploration and expressed as a discrimination index (DI): (tA_4_ – tA_3_) / (tA_4_ + tA_3_) × 100%, where t indicates the time spent exploring the designated object. Memory for the original training information was determined via comparison of exploration of the A_4_ location to locations A_1_ and A_2_ and expressed as a DI to compare exploration of the novel location to objects in the original training locations A_1_: DI = (tA_4_ – tA_1_) / (tA_4_ + tA_1_) × 100%. N = 13-16 mice per group.

***Novel Object Recognition (NOR)***

NOR relies on intact hippocampal, medial prefrontal cortex (mPFC) and perirhinal cortex function and was conducted as previously published (1-3) and beginning ~2.5 months following the completion of GCR exposure after having given the mice at least 2 days of rest following the end of OUL testing. The NOR task measures the preference for novelty as an indicator for episodic recognition memory. Briefly, mice were initially habituated to the empty arena for 3 days (5 minutes per day), and the following testing day mice were allowed 5 minutes to explore 2 identical plastic objects. The mouse was returned to its home cage for 5 minutes during which time 1 familiar object was replaced in the arena with a novel object of different color and shape from the familiar object. The DI for this task was calculated for each mouse after the mouse was allowed another 5 minutes of exploration in the arena. The DI was calculated as: [(novel/total exploration time) – (familiar/total exploration time)] × 100. N = 13-16 mice per group.

***Light-Dark Box (LDB) Test***

We evaluated anxiety-like behavior with the LDB test using established methods and conditions (1, 4) after the mice had rested for at least 2 days following the completion of NOR testing. The arena consisted of a light compartment connected to a dark compartment (30 × 20 × 27 cm, 915 lux vs. 15 × 10 × 27 cm, 4 lux) via a small opening (7.5 × 7.5 cm). As such, the test contrasts the natural propensity of mice to explore new environments with their fear of well-lit open spaces. During 5 minutes of exploration the time a mouse spent in each chamber was recorded as were the number of transitions between compartments. Minute 0-1 served as an acclimation phase and exploration was scored for minutes 1-5. The data presented are for minutes 1-3, where mice were most active. N = 15-16 mice per group.

***Social Interaction Test (SIT)***

Social interaction and avoidance behaviors were evaluated in the same mice that underwent OUL, NOR, and LDB testing using established protocols (1, 5-7) at ~3.5-4 months post GCR Sim exposure. All experimental mice were individually habituated to a well-lit test arena (915 lux, 30 × 30 cm). On the day of the SIT, a novel C57BL/6J mouse of the same sex and weighing less than the test mouse was allowed to explore the arena freely for 10 minutes, after which time the test mouse was placed into the arena. The 2 mice were allowed to explore and interact without barrier for 10 minutes. Video recordings were then scored for active interaction where the test mouse sniffed while in active contact with the novel mouse’s snout, flank, or anogenital area, mutual grooming, or in directed pursuit of the novel mouse. Avoidance behavior was characterized as time the test mouse spent actively avoiding social interactions initiated by the novel mouse. N = 15-16 mice per group.

***Fear Extinction (FE) Testing***

To test whether mice could learn and later extinguish conditioned fear responses, we performed the FE test based on established and published protocols (1, 11) and conducted following SIT training starting at ~4.5 months after the completion of irradiations. Briefly, testing occurred in 2 contexts. The first, context A, was a behavioral conditioning chamber with steel slat floors (3.2 mm diameter slats, 8 mm spacing; 17.5 × 17.5 × 18 cm, Coulbourn Instruments), scented with 10% acetic acid in water. In the second, context B, the steel floor of the chamber was covered with white plastic and the chamber scented with 10% almond extract in water. On day 1 fear conditioning was performed in context A after a 2 minute habituation. Three pairings of an auditory conditioned stimulus (CS) were played co-terminating with a foot-shock unconditioned stimulus as described in Alaghband *et al.* (1). During the following 3 days of extinction training, mice were habituated to context B for 2 minutes before being presented with 20 non-US reinforced CS tones. On the final day fear testing occurred in context B where mice were presented with only 3 non-US reinforced CS tones at 2 minute intervals. Freezing behavior was recorded by overhead chamber cameras and scored by an automated, video-based, motion detection program where a motion index was calculated for each frame of the video and higher indices represent greater movement (FreezeFrame, Coulbourn Instruments). An investigator blinded to the experimental groups set the motion index threshold for each animal individually, based on identifying a trough separating low values during immobility and higher motion-related values. Freezing behavior was defined as continuous bouts of 1 second or more of immobility. The percentage of time each mouse spent freezing was calculated for each phase of testing. Data from the extinction training days are presented as the averages of the first 10 non-US reinforced CS tones per group. FE test data are presented as the average of the three non-US reinforced CS tones for each group. N = 15-16 mice per group.

***Barnes Maze***

Spatial learning was evaluated using the Barnes Maze assay beginning 3 months post-irradiation in a separate group of mice at Harvard University. The apparatus used was similar to that originally described by Barnes (9). The maze platform was a white circular, 0.9 m in diameter, with 20 evenly spaced holes (5 cm diameter), 3 cm in from the edge. The platform was suspended approximately 1 m off the ground, to discourage spontaneously jumping from the platform to the ground. A hidden darkened escape chamber similar in texture to the mouse’s home cage was maintained under 1 of the holes at the edge of the platform. This escape chamber remained oriented in a constant position relative to the room throughout testing, although the platform itself was rotated so as to confuse any possible scent trails. Both the maze and escape box were cleaned thoroughly with 70% ethanol after each trial. Mice were habituated to the testing room conditions for an hour before each day’s training or testing sessions. A camera was positioned above the platform and paired to TopScan tracking software (CleverSys Inc.), which recorded the animal’s position and the distance moved. For each group of animals, the protocol occurred over a 5 day period, with four days of training followed by a single testing day. At the start of day one, mice were first primed for the study by being allowed to explore the maze for 90 seconds. Mice were then guided to the exit hole and placed in the covered escape box for 2 minutes before being returned to their home cage. The training period consisted of 4 trials per day across days 1 to 4. Each trial began by placing the animal under a bucket in the center of the circular platform in a brightly lit room rich in consistently located spatial cues (10). When the bucket was lifted, the mouse was monitored as it found its way from the center of the platform to the escape hole. For each trial, the mouse was given 3 minutes to explore. If the mouse successfully found and entered the exit hole, it was rewarded by having the investigator placing a cover over the hole, allowing the mouse to remain in the box for 60 seconds before returning to its cage. If the mouse did not find the hole within 3 minutes, it would be guided to the location and then allowed 60 seconds of covered time in the box similar to successful trials. A minimum of 15 minutes was allowed between any consecutive trials for individual mice. Time elapsed before the mouse found the hole was recorded for each trial (maximum being 180 seconds for mice who did not find the hole in time). On day 5, the escape box was removed, leaving 20 identical holes. Mice were allowed 90 seconds of recorded exploration, and percent time spent at the target hole and latency to reach target hole was analyzed and recorded. N = 12-14 mice per group.

***Open Field Testing (OFT)***

Following completion of Barnes Maze testing, OFT was performed beginning ~3.5 months post-irradiation. The open field box arena was 50 × 50 cm and the mice were brought into the testing room to acclimate for at least 1 hour before testing began. The 1 hour test session was recorded by a camera mounted directly above the arena and TopScan tracking software (CleverSys Inc.) was used to calculate the percent time each mouse spent in the center of the arena and total distance that they traveled (mm). Each arena was cleaned thoroughly with 70% ethanol after each trial. N = 9-13 mice per group.

***Tube Dominance Test***

For the tube dominance behavioral test, a clear polycarbonate tubing with an inner diameter of 3.5 cm and a length of 30 cm was used to evaluate dominance behaviors beginning 4.5 months following completion of irradiations, using the same mice as those that underwent Barnes Maze and OFT testing and established protocols (8). Mice were paired against one another in a round-robin style. Each mouse faced every other opposing group mouse in what was termed a match. For the mouse pair matches, 8 control and 8 acute GCR experimental mice were initially selected such that their body weights were all within 15% of one another. Every match began by placing each of the 2 mice in separate lidless holding cages with only bedding and on the left and right ends of the dominance tube. A trial ended when a mouse backed all 4 limbs out of the tube and the mouse that was first to leave the tube would lose the trial. Between trials, the tube was cleaned with 70% ethanol. To establish wins, the best 2 of 3 trials were taken. Each trial won scored a point for the winning mouse (maximum total points per match being 3 with 1 mouse earning 2 points and the other earning 1). Two wins in a row resulted in the winning mouse earning 2 points and the losing mouse earning 0. Eight matches total were conducted, with a maximum of one pairing per mouse, per day. N = 8 mice per group.

***Behavior Statistical analyses***

Statistical analyses for the FE test were carried out using GraphPad Prism (v8) software. Conditioning day 1 (T1-T3) and extinction training days were analyzed using two-way ANOVA followed by the Bonferroni’s post hoc test with the percent time spent freezing as within-subjects variables and group/irradiation treatment (*i.e.* chronic vs. acute vs. control). This statistical test was used to make specific comparisons when significant interactions and/or main group effects were observed. For Barnes Maze and OFT, unpaired Student’s t-tests were conducted. As appropriate, behavior data were tested for confirmation of normal Gaussian distribution prior to statistical testing. For these behavior tests, an outlier was defined as a mouse whose behavior was outside of 2 standard deviations of the mean and was excluded from the analysis. Unless stated otherwise, results were expressed as mean ± SEM and all analyses considered a value of *P* $<$ 0.05 to be statistically significant.

**Extracellular field recordings**

Extracellular field recordings were performed at 5 months post-irradiation as described (12). Briefly, isoflurane anesthetized mice were decapitated, the brain quickly removed and submerged in ice-cold, oxygenated dissection medium (in mM: 124 NaCl, 3 KCl, 1.25 KH_2_PO_4_, 5 MgSO_4_, 26 NaHCO_3_, 10 glucose). Coronal hippocampal slices were prepared using a Leica vibrating tissue slicer (320 µm; Model:VT1000S), before being transferred to an interface recording chamber containing preheated artificial cerebrospinal fluid (aCSF) (in mM: 124 NaCl, 3 KCl, 1.25 KH_2_PO_4_, 1.5 MgSO_4_, 2.5 CaCl_2_, 26 NaHCO_3_, 10 glucose; 31 ± 1°C). Slices were continuously perfused with this solution at a rate of 1.75-2 ml/minute while the surface of the slices were exposed to warm, humidified 95% O_2_/ 5% CO_2_. Recordings began after a minimum of 2 hours incubation.

Field excitatory postsynaptic potentials (fEPSPs) were recorded from CA1b stratum radiatum apical dendrites using a single glass pipette in response to orthodromic stimulation of Schaffer collateral-commissural projections in CA1 stratum radiatum (12). Pulses were administered at 0.033 Hz using a current that elicited a 50% maximal spike-free response. A 20-minute stable baseline was established and then long-term potentiation (LTP) was induced by delivering 5 theta bursts, each consisting of 4 pulses at 100 Hz and the bursts themselves separated by 200 ms (i.e., theta burst stimulation; TBS). The stimulation intensity was not increased during TBS. Text data are presented as mean ± SD, figure data as mean ± SEM. The fEPSP slope was measured at 10–90% fall of the slope and data in LTP figures were normalized to the last 20 minutes of baseline. N = 6 mice per group; 1 slice per hemisphere per mouse.

**Whole cell electrophysiology**

Mouse brains were prepared as previously described (12). Briefly, from 2-4 months post-irradiation, male mice were deeply anesthetized by Ketamine/Xylazine and then transcardially perfused with an ice-cold protective recovery solution containing (in mM): 92 NMDG, 26 NaHCO_3_, 25 glucose, 20 HEPES, 10 MgSO_4_, 5 Na-ascorbate, 3 Na-pyruvate, 2.5 KCl, 2 thiourea, 1.25 NaH_2_PO_4_, 0.5 CaCl_2_, pH of 7.3-7.4. Coronal slices containing the hippocampus were cut in the recovery solution using a vibratome and then incubated at 35°C for 12 minutes (300 µm; VT1200S, Leica Biosystems). Subsequently, brain slices were maintained in room temperature aCSF (in mM: 126 NaCl, 26 NaHCO_3_, 10 glucose, 2.5 KCl, 2 MgCl_2_, 2 CaCl_2_, 1.25 NaH_2_PO_4_). All solutions were equilibrated with 95% O_2_/5% CO_2_.

Intracellular recordings were performed as described in Klein *et al.* (12). Briefly, a submerged chamber perfused with oxygenated aCSF at 2.5 ml/min was maintained at 33°C by a chamber heater (BadController V, Luigs and Neumann). Supramammillary neurons were visualized using DIC illumination on an Olympus BX61WI microscope with an sCMOS camera (Olympus Microscopy; Flash 4.0 LT+, Hamamatsu). Recording pipettes with 2.5-5 MΩ tip resistance were pulled from thin-walled borosilicate capillary glass using a P97 puller and filled (in mM: 126 K-gluconate, 10 HEPES, 4 KCl, 4 ATP-Mg, 0.3 GTP-Na, 10 phosphocreatine, pH- 7.3, osmolarity 290 mOsm).

Whole cell recordings were performed on CA1 superficial layer pyramidal neurons in the dorsal hippocampus described (12). Briefly, firing properties were assessed during current injection steps from -100 to 350 pA over 1 second. Exclusion criteria for recordings were defined as neurons with a resting membrane potential above -50 mV or where the series resistance increased by >20% of baseline. Pipette capacitance was neutralized for all recordings. Input resistance, sag, action potential threshold, and width were as described (12). Action potential properties were only measured in the first spike evoked by a depolarizing current for each neuron. Spontaneous excitatory postsynaptic current (sEPSC) activity was measured as inward currents while neurons were held at -65 mV, whereas spontaneous excitatory postsynaptic currents (sIPSCs) were outward currents observed in neurons held at 0 mV. Charge transfer was calculated by integrating the area of postsynaptic currents and rise time was the time required to increase from 10% to 90% of peak amplitude. Events with a rise time > 7.5 ms, a peak amplitude of < 3 pA or a charge transfer of < 25 pC were excluded.

Data were acquired in pClamp software using a Multiclamp 700B amplifier, low-pass filtered at 2 kHz, and digitized at 10 kHz (Molecular Devices). Data analysis was performed using Clampfit (Molecular Devices) or custom written Python and R scripts. Recordings were performed in brain slices obtained from 4-5 mice per group and = 9-14 cells per experimental group.

***Ultrastructural analysis of synapses and myelin***

At 5 months post-irradiation, mice were deeply anesthetized using isoflurane and euthanized via intracardiac perfusion using 1% PFA in PBS (pH 7.4) followed by 4% PFA/0.125% glutaraldehyde in PBS, as described previously (13, 14) and sent to the Icahn School of Medicine at Mount Sinai (ISMMS). EM tissue preparation was performed at The Microscopy CoRE and Advanced Bioimaging Center at the ISMMS. Briefly, the brains were sectioned on a Vibratome (Leica VT1000S) at 250 μm thickness and the CA1 region of the hippocampus, the medial prefrontal cortex (mPFC) and the corpus callosum were isolated. Blocks then underwent cryosubstitution and low-temperature embedding as previously described (13, 14). For synapse analysis, five serial ultrathin sections (80 nm) were collected on formvar/carbon-coated nickel slot grids (Electron Microscopy Sciences) and imaged at USUHS on a JOEL JEM-1011 transmission electron microscope (JEOL USA Inc.) using an AMT XR50S-A camera (Advanced Microscopy Techniques). For myelin analysis, single ultrathin sections (80 nm) were collected on formvar/carbon-coated nickel slot grids (Electron Microscopy Sciences) and imaged at ISMMS on a Hitachi H-7500 transmission electron microscope using an NANOSPRT camera (Advanced Microscopy Techniques).

For synapse analysis in the *stratum radiatum* region of the CA1, 9 image stacks were randomly selected and imaged at a magnification of 15,000×. For the mPFC, 6 image stacks were randomly selected from layer II/III and imaged at a magnification of 10,000×. An unbiased stereological approach using the physical dissector was performed to measure axospinus synapse density as described in our previous work (13, 14). The criteria for inclusion as an axospinous synapse included the presence of a presynaptic terminal and a distinct PSD separated by a clear synaptic cleft. All axospinous synapses were identified within the first two and the last two images of each five-section serial set and counted if they were contained in the reference image (image 1 and 5) but not in the corresponding look-up image (image 2 and 4). To increase sampling efficiency, the reference image and look-up image were then reversed; thus, each animal included in the current study contributed synapse density data from a total of 18 disector pairs. Synapse density was calculated as the total number of unique counted synapses divided by the total volume of the dissector (area × height of dissector). The dissector area for CA1 was 90.969 μm^2^ and the dissector height was 0.16 μm. For the mPFC, the dissector area was 204.677 μm^2^ and the dissector height was 0.16 μm. The same volume was sampled for each group. In addition to total synapse density, we also measured the densities of nonperforated and perforated synapses which were defined by the presence of a discontinuity in the PSD. Serial sections of electron micrographs were manually adjusted for brightness and contrast, and morphological analysis was performed with the aid of Photoshop CC (version 2018, Adobe). A single person, blinded to each of the treatment groups, performed all analyses. N = 5 mice per group.

To characterize the degree of myelination, we calculated the percent of myelinated axons from 12 randomly selected, nonoverlapping fields of the corpus callosum. Cross-sections from each animal were imaged at 1,800×. An additional six randomly selected, nonoverlapping images were taken at 6,000× to evaluate myelin sheath thickness through g-ratio analysis. To calculate the g-ratio, the average diameter for each axon was divided by the average axon diameter plus twice the average myelin width (13-15). Myelin regions that exhibited fixation artifacts or noncompaction were excluded from the analysis. A single person, blinded to each of the treatment groups, performed all analyses. N = 5 mice per group.

**Supplemental Results**

The same male mice that were assessed using the tube dominance test (**Fig. 3D**) were also evaluated using the Barnes Maze spatial memory task and the Open Field Test to measure anxiety (**Supp. Fig. 1**). With regards to the Barnes Maze test, no group differences were observed between control animals and chronic GCR exposed mice, as measured by percent time at target hole or target hole latency (**Supp. Fig. 1A**; t-test: t_(24)_ = 0.28; *P* = 0.79; t_(22)_ = 1.13; *P* = 0.27; respectively). Additionally, no effect of chronic GCR Sim exposure was seen on Open Field Test performance as measured by the percent time spent in the center of the arena of the total distance traveled (**Supp. Fig. 1B**; t-test: t_(20)_ = 0.094; *P* = 0.93; t_(20)_ = 0.51; *P* = 0.62; respectively).

We also examined how the extinction of fear memory, the active process of dissociating learned responses to prior adverse events (10), was altered by chronic or acute GCR exposures in both female and male mice. Mice were subjected to a rigorous protocol designed to elucidate whether they could extinguish the learned behavior of associating a tone with a mild foot shock, an indicator of memory consolidation (**Supp. Fig. 2**). With regards to females, in the conditioning phase all groups were given 3 tone-shock pairings and no interaction or main group effect of treatment was observed (**Supp. Fig. 2A**; T_1_-T_3_; two-way ANOVA: F_(4, 135)_ = 1.84, *P* = 0.13; F_(2,135)_ = 1.35, *P* = 0.26; respectively). During the subsequent fear extinction trials in a new context, all female mice exhibited similar freezing behavior over the course of training with no significant interaction or main treatment group effect observed (Day 1-3; two-way ANOVA: F_(4, 90)_ = 0.48, *P* = 0.75; F_(2, 45)_ = 0.89, *P* = 0.42; respectively). On the final day, an extinction test was given in which only three tones were administered at 2 minute intervals. No differences based on GCR Sim exposure were observed among the female groups on this final extinction test (**Supp. Fig. 2a1**; one-way ANOVA: F_(2, 45)_ = 0.80, *P* = 0.46). Similarly, all male treatment groups show comparable levels of elevated freezing during fear conditioning phase with no significant interaction or main group effect (**Supp. Fig. 2B**; T_1_-T_3_; two-way ANOVA: F_(4, 135)_ = 0.038, *P* = 0.99; F_(2,135)_ = 0.51, *P* = 0.60; respectively). Also, similar to females, during subsequent fear extinction trials in a new context all males exhibited similar freezing behavior over the course of extinction training with no signification interaction or main group effect (Day 1-3; two-way ANOVA: F_(4, 90)_ = 0.85, *P* = 0.5; F_(2,45)_ = 0.094, *P* = 0.91; respectively). Further, when irradiated male mice were given the fear extinction test 24 hours after the cessation of the extinction training trials, no group differences were observed (**Supp. Fig. 2b1**; one-way ANOVA: F_(2, 44)_ = 1.17, *P* = 0.32). Thus, unlike the OUL and NOR assays, FE testing did not suggest impairments in memory consolidation elicited by exposure to chronic or acute GCR Sim.

We also assessed synapse density and complexity in the mPFC to see if chronic or acute GCR exposures affects synaptic density and complexity. We did not observe any significant group differences in total synapse density, perforated and non-perforated synapse density (**Suppl.** **Fig. 3A-C**). To address whether the irradiations alter synaptic complexity, we measured maximum head diameter (HD) and maximum post-synaptic density (PSD) length. Again, we did not observe any significant group differences (**Supp. Fig. 3E-L**).

**Supplemental Information References**

1. Y. Alaghband *et al.*, Neuroprotection of radiosensitive juvenile mice by ultra-high dose rate FLASH irradiation. *Cancers* **12**, 1671 (2020).

2. G. R. Barker, F. Bird, V. Alexander, E. C. Warburton, Recognition memory for objects, place, and temporal order: a disconnection analysis of the role of the medial prefrontal cortex and perirhinal cortex. *J Neurosci* **27**, 2948-2957 (2007).

3. G. R. Barker, E. C. Warburton, When is the hippocampus involved in recognition memory? *J Neurosci* **31**, 10721-10731 (2011).

4. M. Bourin, M. Hascoet, The mouse light/dark box test. *European journal of pharmacology* **463**, 55-65 (2003).

5. J. T. Winslow, Mouse social recognition and preference. *Curr Protoc Neurosci* **Chapter 8**, Unit 8 16 (2003).

6. A. Sato, M. Mizuguchi, K. Ikeda, Social interaction test: a sensitive method for examining autism-related behavioral deficits *Protoc. Exch*, 1-9 (2013).

7. L. A. Gunaydin *et al.*, Natural neural projection dynamics underlying social behavior. *Cell* **157**, 1535-1551 (2014).

8. Z. Fan *et al.*, Using the tube test to measure social hierarchy in mice. *Nat Protoc* **14**, 819-831 (2019).

9. C. A. Barnes, Memory deficits associated with senescence: a neurophysiological and behavioral study in the rat. *J Comp Physiol Psychol* **93**, 74-104 (1979).

10. R. Morris, Developments of a water-maze procedure for studying spatial learning in the rat. *J Neurosci Methods* **11**, 47-60. (1984).

11. C. K. Cain, A. M. Blouin, M. Barad, Temporally massed CS presentations generate more fear extinction than spaced presentations. *J Exp Psychol Anim Behav Process* **29**, 323-333 (2003).

12. P. M. Klein *et al*., Acute, low-dose neutron exposures adversely impact central nervous system function. *Int J Mol Sci* **22**:9020 (2022).

13. D. L. Dickstein *et al.,* Alterations in synaptic density and myelination in response to high-energy charged particles. *J Comp Neurol.***526**, 2845-2855 (2018).

14. B. Krishnan *et al.,* Chronic low dose neutron exposure results in altered neurotransmission properties of the hippocampus-prefrontal cortex axis in both mice and rats. I*nt. J. Mol. Sci.* **22** (2021).

15. V. Murcia-Belmonte et al., Anosmin-1 over-expression regulates oligodendrocyte precursor cell proliferation, migration and myelin sheath thickness. *Brain Struct. Funct.* 221, 1365-1385 (2016).

**Supplemental Figure Legends**

**Supplemental Figure 1. Chronic GCR Sim exposure elicits no alterations in Barnes Maze or Open Field Test behavior in male mice at 3-4 months post-irradiation. A)** Chronically irradiated male mice exhibited no impairments in spatial memory as measured by the percent time spent at the target hole or target hole latency using the Barnes Maze task. **B)** Chronically irradiated male mice exhibited no increase in anxiety-like behavior as measured by the percent time in the center of the arena or the distance traveled during open field testing. Data are mean ± SEM (N = 9-14 per group); *P* values derived from unpaired t-test.

**Supplemental Figure 2. GCR Sim exposure has no effect on fear extinction memory processes in female or male mice at 4.5-5 months post-irradiation. A)** Exposure of female mice to either chronic or acute GCR Sim did not impair the acquisition of conditioned fear memories (T1-T3, tone-shock pairings). The time spent freezing over the extinction sessions (Day 1-Day 3, tone only) was similar across groups. **a1)** During the Fear Extinction Test all female groups equivalently abolished fear memories. **B)** Male mice exhibited no group differences in the acquisition of conditioned fear (T1-T3, tone-shock pairings). All male mice showed a gradual reduction in freezing behavior over the extinction sessions (Day 1-Day 3, tone only) and the time spent freezing was similar across groups. **b1)** During the Fear Extinction Test all male groups equivalently abolished fear memories. Data are mean ± SEM (N = 15-16 mice per group); *P* values derived from two-way repeated measures ANOVA followed by the Bonferroni’s multiple comparisons test.

**Supplemental Figure 3. GCR Sim exposure has no effect on synapse density, HD and PSD length in the mPFC in male mice evaluated at 5 months post-irradiation. A)** Total synapse density. **B)** Perforated synapse density. **C)** Non-perforated synapse density. **D)** Representative electron micrograph depicting non-perforated synapses (white asterisks), perforated synapses (arrow heads) and measurements of PSD length (white line) and head diameter (red line). Scale bar = 500 nm. **E)** HD of total synapses. **F)** Perforated synapse HD. **G)** Non-perforated synapses < 0.4 μm and non-perforated synapses > 0.4 μm respectively. **I)** PSD measurements for all synapses. **J)** PSD measurements for perforated synapses. **K)** PSD measurements for non-perforated synapses < 0.4 μm. **L)** PSD measurements for non-perforated synapses > 0.4 μm. n = 4 animals/group. Data are mean ± SEM, one-way ANOVA * *P* < 0.05, ** *P* < 0.01.

**Supplementary Table 1. Intrinsic properties of CA1 pyramidal neurons following GCR Sim exposure.** Whole cell current clamp recordings of CA1 pyramidal neurons from the superficial layer of the dorsal hippocampus were performed 2-4 months following irradiation. Treatment-dependent group differences were evaluated with a Satterthwaite based F-test of the linear mixed-effect model (LMM) regression.


**Supplementary Table 2. Excitatory and inhibitory synaptic inputs to CA1 pyramidal neurons following GCR Sim exposure.** Whole cell voltage clamp recordings of CA1 pyramidal neurons from the superficial layer of the dorsal hippocampus were performed 2-4 months following irradiation. For the sEPSC frequency measurement where the Satterthwaite based F-test of the linear mixed-effect model (LMM) regression was significant, Tukey’s HSD post-hoc results were: *P* = 0.24 control vs chronic; *P* = 0.044 control vs acute; *P* = 0.60 chronic vs acute. (formatted for landscape)
