# Supplementary figures and images for: Galactic cosmic radiation exposure causes multifaceted neurocognitive impairments
Source: Cell Mol Life Sci. 2023 Jan 6;80(1):29. doi: 10.1007/s00018-022-04666-8 (PMC9823026; doi:10.1007/s00018-022-04666-8)

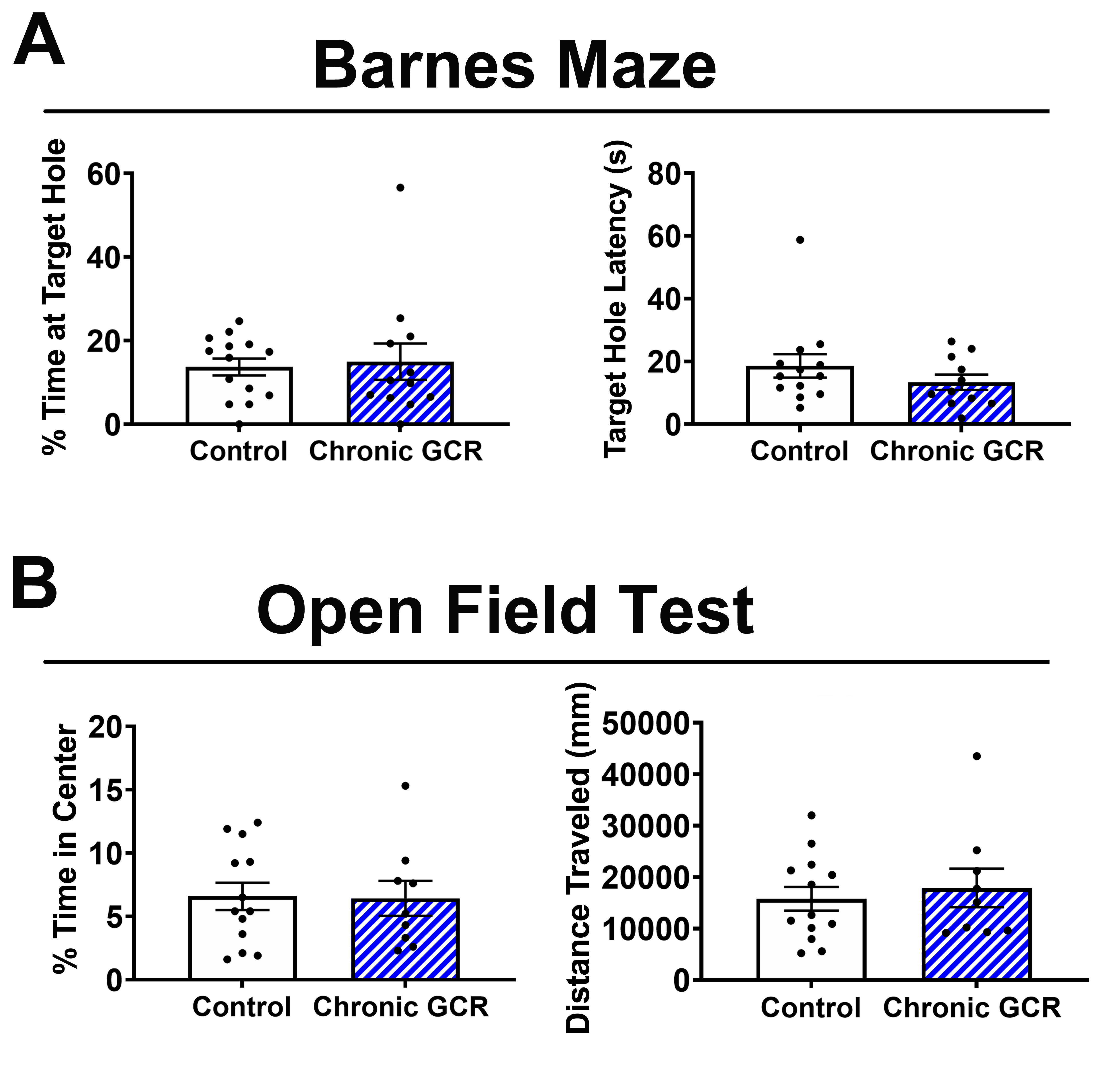

Supplement: Supplementary file 2 — Supplementary file2 (TIF 1385 KB) [file 18_2022_4666_MOESM2_ESM.tif]

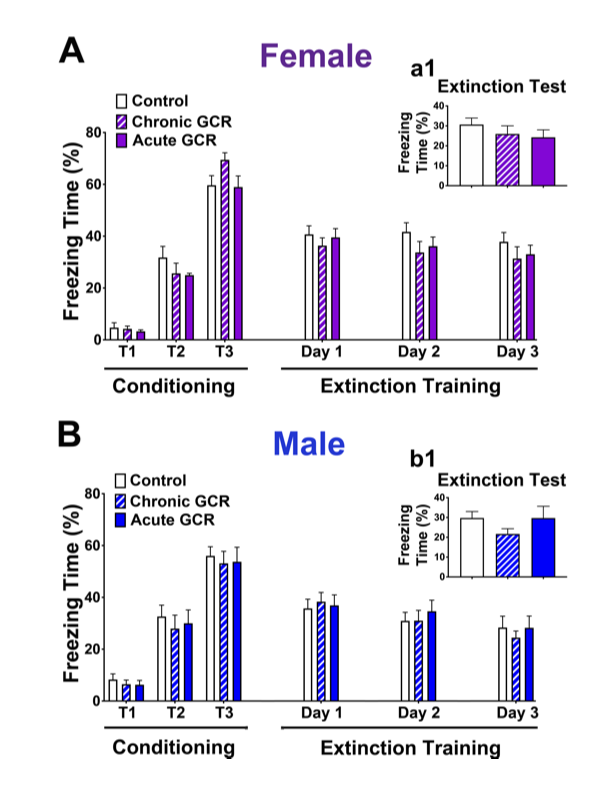

Supplement: Supplementary file 3 — Supplementary file3 (TIFF 1894 KB) [file 18_2022_4666_MOESM3_ESM.tiff]

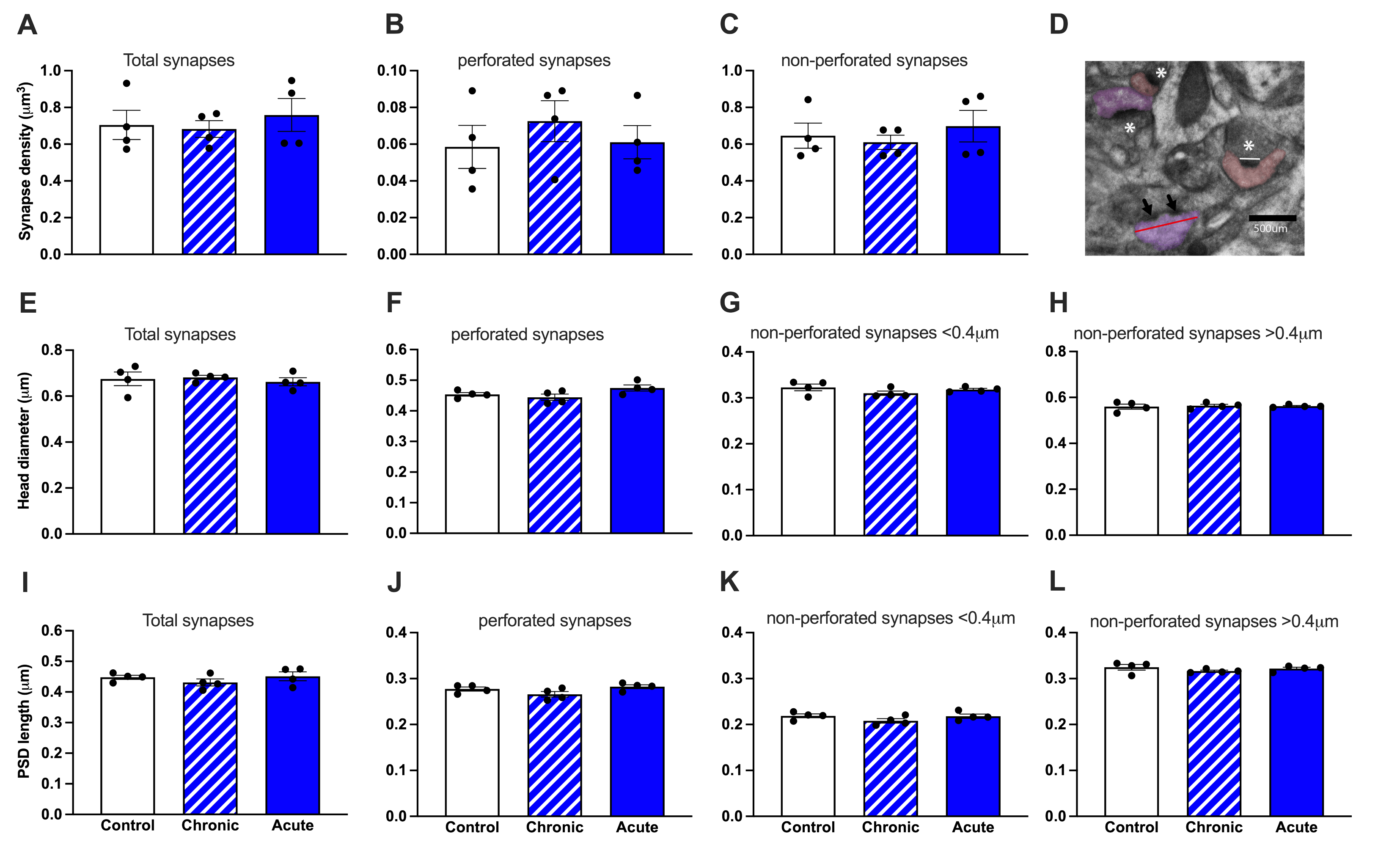

Supplement: Supplementary file 4 — Supplementary file4 (TIFF 1523 KB) [file 18_2022_4666_MOESM4_ESM.tiff]
